# Prevalence of Human Bocavirus in Sewage, Surface Waters, and Other Environmental Milieux: A Meta-regression Modelling

**DOI:** 10.1007/s12560-025-09648-0

**Published:** 2025-06-17

**Authors:** Temitope C. Ekundayo, Frederick T. Tabit

**Affiliations:** Department of Life and Consumer Sciences, University of South Africa, Roodepoort, South Africa

**Keywords:** Wastewater reuse, River, Sludge, Transmission, Groundwater, Lake

## Abstract

Environmental matrices (EMs) are important reservoirs for the human bocavirus (HBoV). This study aimed to determine HBoV prevalence in EMs and model its association with SDG6.3.1 (wastewater production (WWp), collection (WWc), treatment (WWt), and reuse (WWr)). HBoV data-mined from EMs were fitted to a random-intercept-logistic regression/1000-bootstrapped-based meta-regressions. HBoV global prevalence in EMs was 42.19% (95%CI: 28.07–57.72), and varied non-statistically across continents (North America (66.59%), Africa (42.32%), Europe (41.95%), Asia (39.96%), South America (20.55%)), economies (high-income (45.04%) > upper-middle-income (42.56%) > lower-middle-income (41.35%)), WHO regions (Western Europe (41.95%) > Middle East & North Africa (41.35%) > East Asia & Pacific (39.96%) > North America (66.59%) > Latin America & Caribbean (20.55%)) but significantly across dwelling settings (urban: 52.02% versus rural: 20.29%) and sample types (raw sewage (RS, 73.16%) > treated sewage (TS, 43.47%) > sewage sludge (SS, 19.87%) > sediment (13.24%) > surface waters (18.55%)). HBoV subtypes circulating in EMs varied among sample types (HBoV1 in TS (40.74%) > RS (22.45%) > surface water (9.09%); HBoV2 in RS (75.42%) > TS (54.82%) > surface water (18.24%); HBoV3 in RS (64.74%) > TS (58.95%) > surface water (6.48%) > SS (3.49%)). HBoV prevalence in EMs had direct relationship (*p* ≤ 0.05) with SDG6.3.1 variables (WWp: F_1;35_ = 4.5822), and WWr: F_1;35_ = 4.3735; WWt: F_1;35_ = 3.9517; and WWc: F_1;35_ = 3.3510) accounted for 17.13%, 15.79%,15.65%, and 12.92% of the estimate variance, respectively. In conclusion, HBoV prevalence is high in EMs globally, across regions, sample types and showed considerable affinity with SDG6.3.1 variables.

**Supplementary Information:**

The online version contains supplementary material available at 10.1007/s12560-025-09648-0.

## Introduction

Human bocavirus (HBoV) is an emerging gastrointestinal and respiratory infectious agent particularly in the infants and children (Kumthip et al., 2021). The HBoV presence in environmental matrices including river, sewage, lake, sludge, and aquatic sediments is an indication of their roles in the persistence and transmission dynamics coupled with the wide distribution and circulation of HBoV subtypes in the environments (Shaheen et al., 2020). These environmental matrices are potential public health risk since they can harbor and facilitate the transmission of HBoV (Räsänen et al., 2010). While previous studies have documented HBoV presence in different environmental milieu involving sewage, surface water, aquatic sediment, and sewage sludge in Germany (Hamza et al., 2009), Finland (Räsänen et al., 2010), Italy (Iaconelli et al., 2016), Brazil (Salvo et al., 2018), Uruguay (Salvo et al., 2018), Belgium (Rector et al., 2022), Egypt (Shaheen et al., 2020; Hamza & Abd-Elmaksoud, 2023), South Africa (Onosi et al., 2020), Australia (Ahmed et al., 2020), Thailand (Booranathawornsom et al., 2022), Spain (Mejias-Molina et al., 2024), and China (Peng et al., 2024), environmental milieu have not been demonstrated as transmission route of HBoV infection in humans. However, the abundance of HBoV in treated/raw sewage and environmental matrices suggests their potential contribution in its dissemination among human population (Peng et al., 2024) and thus should not be overlooked. In addition, the presence of HBoV in the environmental milieu may pose human infection and ecological risks, but it is also critical to its transmission across the food supply systems. For instance, sewage discharge into waterbodies creates an avenue for shellfish contamination by HBoV and attending human infection via consumption of contaminated shellfish. Shellfish being filter-feeders concentrate and biomagnified HBoV in aquatic milieu (Purpari et al., 2019), HBoV has been detected in mussels, bivalve, oysters, and clams in Thailand (Kumthip et al., 2021), Italy (La Rosa et al., 2018; Purpari et al., 2019), Brazil (Nascimento et al., 2022), South Africa (Onosi et al., 2020) and Brazil (Nascimento et al., 2022) to mention a few.

The SDG 6.3.1 targetted at improving treated (waste)water quality either by eliminating, reducing or minimizing biological and chemical contaminants to ascertain its safe re/use for potable or irrigation purposes without any direct or indirect risk (Nations, 2015). Regardless of improvements in modern waste(water) treatment technologies, the presence and persistence of HBoV in raw and notably in treated (waste)water and environmental waters pose substantial challenges (Räsänen et al., 2010; Shaheen et al., 2020; Peng et al., 2024). HBoV, primarily known to manifest respiratory and gastrointestinal infections (Rikhotso et al., 2018; Bergallo et al., 2019), has been increasingly detected in raw and treated wastewater matrices (Kumthip et al., 2021). Thus, recycled water directed for human consumption or agricultural applications raise vital public health concerns as HBoV environmental stability, transmission potential, and fate during treatment processes are unknown. The knowledge gap hinges on the deficient HBoV global/regional prevalence and distribution patterns data in environmental waters and milieu. This constraint hinders HBoV risk assessment and the formulation of guidelines crucial for protecting public health under SDG 6.3.1. Additionally, conventional (waste)water treatment and reuse risk management practices frequently do not consider the persistence of emerging biological contaminants like HBoV (Baggi et al., 2001; Hot et al., 2003; Gerba et al., 2013), thereby threatening the safe re/use of treated (waste)water for potable and agricultural purposes. The traditional reclaimed water re/use microbiological risk management practices often solely relied on indicator bacteria and bacteriophages whose presence do not always predict the occurrence or fate of viral pathogens in environmental waters (Baggi et al., 2001; Hot et al., 2003; Gerba et al., 2013).

Summarily, there is a critical paucity of comprehensive data on HBoV prevalence, their infectivity and distribution patterns in environmental milieu at regional and global resolution. This lack of HBoV prevalence and distribution pattern data at regional scale, particularly hampers the ability to evaluate its regional risk factors and to formulate localized management strategies. Also, mainstream (waste)water treatment techniques or protocols may not consistently remove or inactivate HBoV, the discharge/reuse of which could lead to surface waters, aquatic products like shellfish, and agricultural products contamination. This contamination can hence facilitate HBoV transmission to humans through direct contact or contaminated water/shellfish, and agricultural produce consumption (Kitajima et al., 2011; Purpari et al., 2019; Nascimento et al., 2022). The recycling of treated (waste)water for potable and irrigation uses without a well-defined and comprehensive grasp of the specifics related to HBoV contamination levels increases the risk of transmission. This exposure could have considerable consequences, especially in regions where water recycling is an integral component of water resource management (Kitajima et al., 2011).

Hence, this study aimed to synthesize the global prevalence of HBoV and HBoV subtypes in environmental milieu and decompose it by region, sample type, and dwelling setting. Furthermore, to identify the association between HBoV prevalence in environmental milieu and SDG 6.3.1 (wastewater production ($W{W}_{p}$), collection ($W{W}_{c}$), treatment ($W{W}_{t}$), and reuse ($W{W}_{r}$)), technical, and regional (continent, economic grouping and WHO classification) variables aimed at indirect assessment of HBoV risk associated with $W{W}_{p}$, $W{W}_{c}$, $W{W}_{t}$, and $W{W}_{r}$ of raw and treated environmental waters, sewage, and sludge. The study provides invaluable data on environmental persistence of HBoV.

## Materials and Method

### Study Design

The targeted sample matrix types included sewage, river, stream, lake, groundwater, pond, estuary, dam, sludge and sediment. The outcome of interest was the presence or absence of either infectious or non-infectious HBoV particles in the matrices. Summarily, the study design relied on the Population, Medium, and Outcome (PMO) framework. The study was approved by the College of Agriculture and Environmental Sciences_Health REC, University of South Africa with the ethical clearance reference number 2025/CAES_HREC/6687.

### Study Selection

Basic research data sources on the assay of HBoV in the matrices listed in “Inclusion and Exclusion Criteria” Sect. were mined rigorously from EBSCOhost interfaced with 26 other repositories, Scopus, WoS (Web of Science), and PubMed from inception till Dec 31, 2024 without imposing any restriction in language or location using search words “‘human bocavirus’ AND (water* OR river* OR stream* OR lake* OR groundwater* OR pond* OR estuar* OR dam OR wastew* OR sewage*)” as a topical algorithm in Scopus, WoS, and PubMed, but as all-field algorithm in EBSCOhost. The algorithm was adaptable to the different repositories and detailed in the supplemental material per PRISMA version 2020 (“Preferred Reporting Items for Systematic Reviews and Meta-analyses)” (Page et al., 2021).

### Inclusion and Exclusion Criteria

#### Inclusion Criteria

Any primary data source to be included must satisfy the following listed criteria: (1) It must be an original investigation or assay of HBoV (presence/absence) in matrices listed in “Study selection” section; (2). The technical information associated with sample collection, HBoV concentration, process control, and detection methods must be adequately described; (3). The full text of the primary data source must be available in any language; and (4). In any case where the matrices in “Study selection” section or HBoV was co-concurrently assayed with other samples or viruses, the data specific to listed matrices in “Study selection” section and HBoV was extracted.

#### Exclusion Criteria

Any data source that satisfied at least one of the following criteria was excluded: (1) HBoV was assayed in other matrices other than those listed in “Study selection” section. For example, HBoV in clinical samples, animals or food samples; (2) Stimulated experimental studies; (3) Method development related to HBoV without final assessed on the matrices listed in “Study Design” Sect.; and (4) Primary data source that did not provided HBoV specific information required in sections “Inclusion criteria” and “Data treatments”.

### Data treatments

All primary data sources retrieved from the databases were merged and de-duplicated in Endnote version 21.1 and Excel version 2016. The output records were screened by titles/abstracts. Eligible studies were retrieved for data extraction into excel forms. Targeted data were extracted by TE in two separate trials. Extracted data were validated by taking the difference between the two datasets where a zero output indicated data equality. The co-researcher did a second round of data validation. HBoV presence in an environmental matrix was defined as a nested PCR or real-time PCR detection of HBoV DNA in the matrix. Extracted data included publication year (PY), author, HBoV (P), sample size (N), sampling method, HBoV concentration method, extraction kit, process control means, detection method, nation and locational setting of the study. The validated data was decomposed by the matrix types for grouping and stratification. The quality of the individual primary data sources was thereafter evaluated (Ekundayo et al., 2023).

External data such as countries’ economic classification, SDG 6.3.1 data, and WHO region classification were retrieved from the respected websites. SDG 6.3.1 data included wastewater production ($W{W}_{p}$ million ${m}^{3}$/yr), collection ($W{W}_{c}$ million ${m}^{3}$/yr), treatment ($W{W}_{t}$ million ${m}^{3}$/yr), and reuse ($W{W}_{r}$ million ${m}^{3}$/yr) at the country scale.

### Statistical analysis

Based on the primary data sources, a total of 1857 environmental matrix samples assayed for HBoV were collated and summarized descriptively. The sample matrix types were further disaggregated by sample type with the associated HBoV crude prevalence (%) calculated and tested for bivariate correlation with $W{W}_{p}$, $W{W}_{c}$, $W{W}_{t}$, and $W{W}_{r}$, technical information, economic and WHO classification variables. All prevalence data was logit-normalized (Schwarzer et al., 2019) and fitted to random-intercept logistic regressions with a half (0.5) input for continuity correction according to Eqs. 1 and 2 (Schwarzer et al., 2019).

1 $${\theta }_{s}^{10}=lo{g}_{e}\frac{{p}_{s}}{\left(1-{p}_{s}\right)},{\theta }_{s}^{10}\sim \theta +{\mu }_{s},with{\mu }_{s}\sim N\left(0,{\tau }^{2}\right).$$

2 $${y}_{i}={\beta }_{0}+{\beta }_{1}{x}_{v}+{u}_{v}+{\epsilon }_{v}.$$

where *p* = proportion, ${\beta }_{0}$ = prevalence (overall effect size), ${\beta }_{1}x$ = regression term, ${\mu }_{v}$ = random-effect term with ${\mu }_{v}\sim N\left(0,{\tau }^{2}\right)$, $\beta$ weights = the common effects elements, ${\epsilon }_{v}$ = random error with ${\epsilon }_{v}\sim N\left(0,\sum_{v}\right)$, and the number of events in a study (${\mu }_{s}$) is implied distributed as:

$${\mu }_{s}\sim B\left({n}_{s},\frac{exp\left({t}_{s}^{10}\right)}{1+exp\left({t}_{s}^{10}\right)}\right)$$

The model’s stability or sensitivity was assayed through leave-one-study-out-cross-validation (Viechtbauer & Cheung, 2010). The non-combinability/heterogeneity of data sources was assessed using maximum-likelihood method and reported as ${I}^{2}$ (inconsistency) statistic where an ${I}^{2}\ge 75$ depicted a large variance (Higgins & Thompson, 2002) (Eqs. 3 and 4). Where $Q=\sum_{\left(s=1\right)}^{s}{\omega }_{s}{\left({\widehat{t}}_{s}-\widehat{t}\right)}^{2}$ and ${\widehat{t}}_{s}-\widehat{t}\sim N\left(0,1\right)$,

3 $${I}^{2}=\frac{Q-\left(\left(S-1\right)\right)}{Q}.$$

4 $${H}^{2}=\frac{Q}{\left(S-1\right)}.$$

${\omega }_{s}$ = weighting term; mean $\widehat{t}$ = overall effect in the fixed-effect model; *Q* follow a ${\chi }^{2}$ distribution with S−1 degrees of freedom, where there is no heterogeneity. Small-study bias or effects was assessed via Egger’s regression (Egger et al., 1997). Egger’s Regression Test:

$$\frac{{\widehat{\theta }}_{k}}{S{E}_{{\widehat{\theta }}_{k}}}={\beta }_{0}+{\beta }_{1}\frac{1}{S{E}_{{\widehat{\theta }}_{k}}}$$

${\beta }_{0}$ & ${\beta }_{1}$ = Regression intercept and weight, respectively, SE = standard error. Dataset was further grouped according to sample type, regional classification (continent, setting, economic classification, WHO) and technical information and fitted in a mixed-effects subgroup analytic model where subgroup differences were determined through a common-effects methodology and within-group HBoV prevalence through a random-effects procedure (Borenstein & Higgins, 2013). The subgroup generalized logistic-mixed-effects models is defined as:

$${\widehat{\theta }}_{k}=\theta +\beta {D}_{g}+{\epsilon }_{k}+{\zeta }_{k}$$

$${D}_{g}={\{}_{1:{\widehat{\theta }}_{k}={\theta }_{\rm A}+{\theta }_{\Delta }+{\epsilon }_{k}+{\zeta }_{k}}^{0:{\widehat{\theta }}_{k}={\theta }_{\rm A}+{\epsilon }_{k}+{\zeta }_{k}}$$

where the value of $\beta$ = the effect size difference ${\theta }_{\Delta }$ between subgroup A (0) and subgroup B (1). A series of 1000-bootstrapped meta-regressions, where the inputs were provided as continuous (SDG 3.6.1 data, sample size) or discrete (WHO, economic classification, technical information) variables was fitted to assess their relationship with HBoV prevalence in environmental matrices (Good, 2013; Viechtbauer et al., 2015). The uni(multi)variate meta-regression was defined as:

$${\widehat{\theta }}_{k}=\theta +\beta {x}_{k}+{\epsilon }_{k}+{\zeta }_{k}$$

where ${\widehat{\theta }}_{k}$ = overall effect size of study k, ${\epsilon }_{k}$ = the sampling error, through which the effect size of a study deviates from its true effect. ${\zeta }_{k}$ = true effect size of the study from an overarching effect sizes distribution. The meta-regression model will be fitted using meta r package in the form: ${y}_{i}\sim A$ in univariate and as ${y}_{i}\sim A+B+\dots +\dots$ in multivariate model, where A, B, and $\dots$, are the variables in the study; ${y}_{i}={\widehat{\theta }}_{k}$. All models and analyses were implemented in 4.4.2 (2024-10-31 ucrt).

## Results

**Fig. 1:**
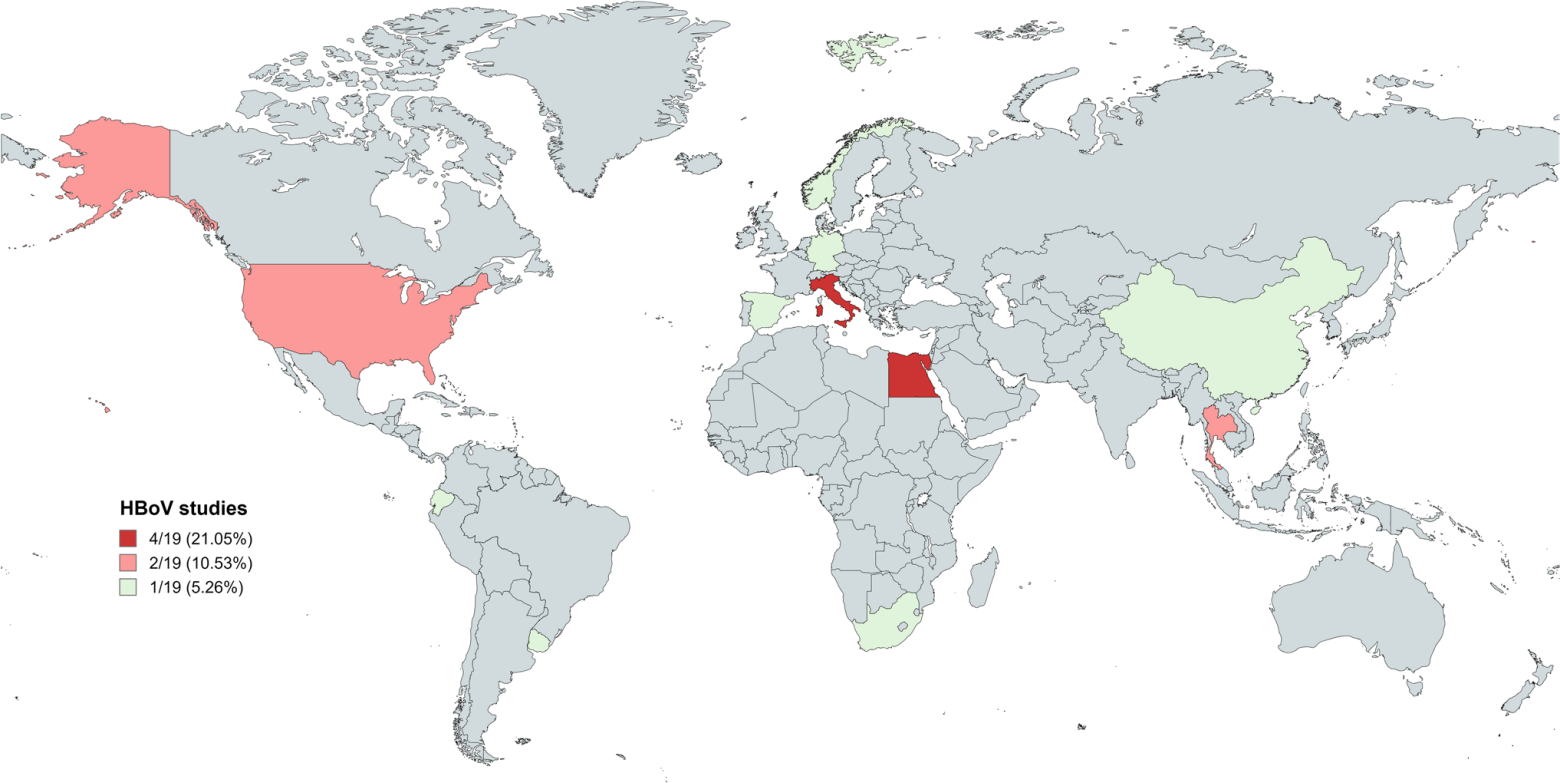
Geographical distribution of studies on HBoV in sewage, surface waters and other environmental matrices

A total of 104 data sources were identified from the database using the search words and 36 data sources were retained after removing duplicates. These 36 data sources were screened using the inclusion criteria and 20 data sources were retained for systematic data extraction. However, 19 data sources were included in the final quantitative synthesis as disaggregated data based on the sample matrix types (*n* = 37) (Fig. S1a). Figure 1 presents the geographic distribution of the 19 data sources on HBoV prevalence in sample matrix types; and were mainly from Egypt, Italy, Thailand, USA, China, Ecuador, Germany, Norway, South Africa, Spain, and Uruguay in descending order.

Tables 1 and 2 respectively present the main overview of the 20 data sources and explanatory characteristics of the 37 sample-based disaggregated datasets. A total of 1857 samples from the 19 data sources with an overall average sample size of $50.19\pm 44.99$ per 37 disaggregated data sources were found. The mean HBoV positivity per 37 disaggregated data sources was $21.54\pm 24.58$. The representative environmental sample included raw sewage (15/37), surface water (9/37), treated sewage (7/37), sediment (3/37), and sewage sludge (3/37) sampled either via grab (30/37), composite (5/37), or swab (1/37) sampling method. HBoV concentration method in the samples varies from the use of adsorption–elution method, adsorption–elution with membrane filtration, centrifugation–dextran–PEG, centrifugation–glycine–CF, centrifugation–polyethylene glycol, negative filter, negative filter–adsorption–elution–PEG, negative filter–adsorption elution, negative filter–centrifugation–adsorption–elution method, negative filter–organic flocculation, polyether sulfone membrane filter cartridge, and ultrafiltration–adsorption–elution methods. HBoV concentration process control were achieved via the use of Mengovirus (5/37), murine norovirus (8/37), Simian rotavirus–human adenovirus type 2 (7/37), known viral positive sample (6/37), human adenovirus type 2 (5/37), Pepper mild mottle virus (1/37), and process control was not reported in 5/37 sample sources. HBoV was detected in the environmental samples through nested PCR (27/37) and real–time PCR (10/37). While 27/37 (73%) of the environmental matrix samples were from urban settings, only 9/37 (24%) were from rural settings. Also, the data sources had different regional distribution in term of Continent (Africa (17/37), Asia (6/37), Europe (9/37), North America (3/37), South America (2/37)), WHO region (East Asia & Pacific (6/37), Latin America & Caribbean (2/37), Middle East & North Africa (16/37), North America (3/37), Sub–Saharan Africa (1/37), and Western Europe (9/37)), and economic classification (high income (14/37), lower middle income (16/37), and upper middle income (7/37)).

**Table 1:** Detail information on the included primary data sources on HBoV prevalence in environmental matrices

Author	Study.period	P	N	Sample	Stype	S.preparation	Concentration.mtd
Myrmel et al. (2015)	February 2008–February 2009	52(30//22)	102 (51/51)	Raw/Treated Sewage	Composite	Untreated sewage samples were centrifuged at 43009 g/30 min at 4 C	Polyethylene glycol (PEG) 8000 and NaCl to a final concentration of 8% (w/v)and 17.5%(w/v); centrifugation (10,0009 g /90 min at 4 °C)
Hamza and Abd-Elmaksoud (2023)	08/2018 and 07/2019	43	45	influent/effluent wastewater	Grab	Negatively charged 0.45um HA nitrocellulose membrane	Organic flocculation technique
Kumthip et al. (2021)	November 2016 to July 2018	34	126	Environmental waters((env.reservoir 2/21; garden4/21); irrigation canal11/42; river3/21; wastewater 14/21)	Grab	Centrifuged at 10,000 g/30 min at 4 °C	Polyethylene glycol (PEG) precipitation
Crank et al. (2020)	2014 to 2017	113 (GCF (83/114)/ PEG-dextran (21/29))	156	Wastewater influent	Grab (126)/ composite (30)	Centrifugation 2300 g /10 min	glycine-CF(chloroform and centrifuged) method/ dextran T40 and polyethylene glycol (PEG) 6000
Hamza et al. (2009)	January–December 2008	49	120	River	Grab	HA negatively charged membrane filter 0.45um	modified adsorption–elution method, non-organic elution buffer PEG-6000 precipitation
Shaheen et al. (2020)	April 2017 to March 2018	RP	RP	Untreated raw sewage (5/12), treated raw sewage (3/12), sewage sludge (2/12), drainage water (35/72), Drain sediment (7/24), river water (3/24), and river sediment (1/24)	Ns/grab	Adsorption–elution method, using a negatively charged membrane	PEG 6000 precipitation method
Iaconelli et al. (2016)	2014 to 2015	106	134	Raw sewage	Grab	Centrifuged at 5000 rpm for 10 min	Glycine-CF
Blinkova et al. (2009)	2007	17	21	Raw sewage	Grab	0.45- m polyether sulfone membrane filter cartridge	NR
Salvo et al. (2018)	March 2011 to February 2012//September 2011 and April 2013// June 2015 and May 2016	(47/68//1/36)	104(68/36)	Sewage/surface Waters	Composite/grab	NR	Adsorption-elution to negatively charged membrane
Peng et al. (2024)	January to December 2021	55//57	57	Wastewater	Grab	10% PEG 8000 and 0.25 mol/l NaCl for 30 min; centrifuged at 7000 g for 30 min at 4.C	Polyethylene glycol (PEG) precipitation
Booranathawornsom et al. (2022)	2007 to 2020; 14 years)	9//27/86	102//86	Recycled water/sewage sludge	Grab	NR	Adsorption-elution technique with membrane filtration/ adsorption-elution method
Purpari et al. (2019)	NR	0	23	23 water samples (seawater, pipe water, and torrent water)	Grab	Membranes (SG Hydrosart 10 kDa) pre-treated with 300 ml of 3% beef extract, pH 7, 10 min (pressure) ≤ 1.30 bar	Tangential ultrafiltration system
Badr et al. (2024)	December 2019 to November 2021	RP	264	6/24 untreated, 4/24 treated, 2/24 sludge sewage// 20/96 drain water, 11/96 drain sediment	Grab	Negatively charged membrane (H2SO4; 0.45 µm// centrifugation at 2,422 g/15 min at 4 °C, glycine buffer (0.25 M) at pH 9.5// centrifugation at 7,656 g/30 min at 4 °C	Adsorption–elution method//PEG 6000 precipitation method, glycine-chloroform-centrifugation
Guerrero-Latorre et al. (2018)	June 2017,	HBoV qualitatively	3	River	Grab	Centrifuged at 8000 × g for 40 min,	Skimmed Milk Flocculation
Hamza et al. (2017)	2014–2015	62(34//28)	66(34//32)	Sewage (influent/effluent): T: 38/66HBoV1, 61/66HBoV2, 62/66HBoV3	Grab	Filtration negatively charged HA nitrocellulose membrane, 0.45 um,	Virus adsorption-elution method/ organic flocculation
Onosi et al. (2020)	May 2016//	6//10	60 (pooled into 10)	Raw sewage	Swabbing separation grids	Centrifugation at 9000 × g for 3 min	Glycine-cf
La Rosa et al. (2017)	March and October 2014 and April and November 2015	12	32(8 urban)	River	Grab	20-, 5 - and 0.22- um filters	Hollow fiber ultrafiltration approach (UF)/ adsorption-elution-1MDS electropositive filters
Mejias-Molina et al. (2024)	January and March 2022	NA	31	Wastewater (influent/effluent)	3D printed torpedo shaped passive sampling	Two 0.45 μm electronegative membranes, Centrifugation at 20.000 × g/1 min	Adsorption-elution with membrane filtration
Righi et al. (2023)	2022	74	200	Raw sewage	NR	NR	NR

Author	Process.control	Level	Detection	Setting	HBoV.type	DNA.extraction	Target.gene	Nation
Myrmel et al. (2015)	Mengovirus (MC0)	log4.5 gc/l//4.5	Nested PCR	Urban (14/26//17/26 ), rural (16/25//5/25)	NA	NucliSENS easyMAG	VP1/VP2	Norway
Hamza and Abd-Elmaksoud (2023)	Murine norovirus (MNV)		PCR/qPCR	Urban	HBoV1(19/23//11/22; T30/45); HBoV2 (23/23//20/22; T43/45); HBoV3(23/23//20/22; T43/45)	QIAamp DNA Blood Mini Kit	NP1/NS1/NS1	Egypt
Kumthip et al. (2021)	Known HBoV-positive stool samples	NA	Nested PCR	Urban	HBoV1 (8/126), HBoV2 (21/126), HBoV3(2/126), HBoV4(3/126)	Geneaid Viral Nucleic Acid Extraction Kit	VP1/VP2	Thailand
Crank et al. (2020)	Murine norovirus (MNV-1),	To be extract	Nested PCR	Urban/rural	NA	NucliSENS miniMAG semi-automated extraction system	VP1/VP2	USA (sample from Italy)
Hamza et al. (2009)	HBoV-positive sewage sample	3e1 to 2e3 gc/l	Real-time PCR	NR	NA	QIAamp DNA Blood Mini kit	NP1	Germany
Shaheen et al. (2020)	Simian rotavirus and human adenovirus type 2	NA	Nested PCR	Urban	NA	QIAamp Viral RNA and DNA kits	VP1/VP2	Egypt
Iaconelli et al. (2016)	Murine norovirus (MNV1)	5.51e3 to 1.84E5 GC/L	Nested PCR	Urban	HBoV2(49/134), HBoV3 (27/134), HBoV1&2(29/134), HBoV2&4(1/134)	NucliSENS miniMAG semi-automated extraction system	VP1/VP2	Italy
Blinkova et al. (2009)	NR	NA	Nested PCR	Urban	4/67 sequences (6%) HBoV, 45/67 seq.(67%) HBoV2, and 18/67seq. (26%) HBoV3	QIAamp MinElute virus spin kit	VP1/VP2	USA
Salvo et al. (2018)	NR	Mean 8.2e4//7.8e6//4.1e6 gc/l; rR: 2.7e4 HBoV3	Multiplex qPCR/ monoplex qPCR	Urban	HBoV1 (11/104), HBoV2/4 (39/104), HBoV3 (35/104)/WW: 11/68HBoV1; 39/68HBoV2/4;35/68HBoV3	QIAmp Cador Pathogen mini kit	NS1//VP1/VP2	Uruguay
Peng et al. (2024)	Pepper mild mottle virus (PMMoV)	2.54–7.40 log10	Multiplex rt qPCR/ monoplex qPCR/Amplicon-seq	Urban	26/57 HBoV2 24/57 HBoV3 9/57 HBoV1	Viral DNA/RNA kit	VP1/VP2	China
Booranathawornsom et al. (2022)	Norovirus- and/or rotavirus-positive samples	NA	Nested PCR	Urban	26/188 HBoV2; 8/188HBoV3; 1/188HBoV4//ww: 23/68HBoV2, 3/86HBoV3, 1/86HBoV4///Rw:3/102HBoV2, 5/102HBoV3	QIAamp® DNA Mini Kit and/QIAamp®RNA Extraction Kit	VP1/VP2	Thailand
Purpari et al. (2019)	Mengovirus	NA	Nested (RT) PCR	Rural	NA	NucliSENS miniMAG extraction/QIAamp Viral RNA Mini KitQIAamp Viral RNA Mini Kit	VP1/VP2	Italy
Badr et al. (2024)	Human adenovirus type 2	NA	Nested PCR	Rural	NA	QIAamp Viral RNA and DNA kits	VP1/VP2	Egypt
Guerrero-Latorre et al. (2018)	NA	NA	Illumina MiSeq	Urban	NA	Qiagen RNA ViralMini Kit	VP1/VP3	Ecuador
Hamza et al. (2017)	Murine norovirus	Median 8.5e3gc/l HBoV1, 3.0e4 GC/l HBoV2,2.5e4 GC/l HBoV3//2.9e3gc/l HBoV1, 4.1e3 gc/l/l HBoV2, 2.1e3gc/l HBoV3	Real-time PCR	Urban	27/34HBoV1, 34/34HBoV2, 34/34HBoV3 //11/32HBoV1, 27/32HBoV2, 28/32HBoV3	QIAamp DNA BloodMini Kit	NP1/NS1/NS1	Egypt
Onosi et al. (2020)	NA	NA	Nested PCR	Urban	HBoV 3	QIAamp Viral RNA and DNA kits	VP1/VP2	South Africa
La Rosa et al. (2017)	Murine norovirus	NA	Nested PCR	Urban	11/32HBoV2, 1/32HBoV3	NucliSENS easyMAG// NucliSensminiMAG kit	VP1/VP2	Italy
Mejias-Molina et al. (2024)	NA	NA	Illumina NextSeq2000	Urban	HBoV	QIAamp Viral RNA mini kit Qiacube Automatic system	NA	Spain
Righi et al. (2023)	NR	NR	Real-time PCR/Sanger sequencing	Urban	63/200HBoV2	NR	NR	Italy

*NR* not reported, *NA* not applicable, *P* HBoV-positive sample, *N* sample size, *Stype* sample type, S-preparation sample preparation, *mtd* method

**Table 2:** Explanatory characteristics of primary data of HBoV prevalence from 37 sample sources

Characteristic	${K}_{d}$= 37
P	21.54 (24.58)^a^
N	50.19 (44.99)^a^
PR	43.64 (30.70)^a^
Sample type	n/${K}_{d}$ (%)
Raw sewage	15/37 (41)
Sediment	3/37 (8.1)
Sewage sludge	3/37 (8.1)
Surface water	9/37 (24)
Treated sewage	7/37 (19)
sampling method	n/${K}_{d}$ (%)
Composite	5/37 (14)
Grab	30/37 (81)
NR	1/37 (2.7)
Swabbing	1/37 (2.7)
Concentration method	n/${K}_{d}$ (%)
Adsorption–elution method	1/37 (2.7)
Adsorption–elution with membrane filtration	1/37 (2.7)
Centrifugation–dextran–PEG	1/37 (2.7)
Centrifugation–glycine–CF	4/37 (11)
Centrifugation–polyethylene glycol	11/37 (30)
Negative filter	1/37 (2.7)
Negative filter–adsorption–elution–PEG	1/37 (2.7)
Negative filter–adsorption elution	7/37 (19)
Negative filter–centrifugation–adsorption–elution method	3/37 (8.1)
Negative filter–organic flocculation	3/37 (8.1)
NR	1/37 (2.7)
Polyether sulfone membrane filter cartridge	1/37 (2.7)
Ultrafiltration–adsorption–elution	1/37 (2.7)
Ultrafiltration system	1/37 (2.7)
Process control during HBoV concentration	n/${K}_{d}$ (%)
Human adenovirus type 2	5/37 (14)
Known viral positive sample	6/37 (16)
Mengovirus	5/37 (14)
Murine norovirus	8/37 (22)
NR	5/37 (14)
Pepper mild mottle virus	1/37 (2.7)
Simian rotavirus–human adenovirus type 2	7/37 (19)
Detection method	n/${K}_{d}$ (%)
Nested PCR	27/37 (73)
Real–time PCR	10/37 (27)
Setting	n/${K}_{d}$ (%)
NR	1/37 (2.7)
Rural	9/37 (24)
Urban	27/37 (73)
DNA extraction	n/${K}_{d}$ (%)
Geneaid Viral Nucleic Acid Extraction Kit	3/37 (8.1)
NR	1/37 (2.7)
NucliSENS easyMAG	4/37 (11)
NucliSENS miniMAG system	6/37 (16)
QIAamp DNA Blood Mini Kit	5/37 (14)
QIAamp MinElute virus spin Kit	1/37 (2.7)
QIAamp Viral RNA and DNA Kit	14/37 (38)
QIAmp Cador Pathogen mini Kit	2/37 (5.4)
Viral DNA/RNA Kit	1/37 (2.7)
Nation	n/${K}_{d}$ (%)
China	1/37 (2.7)
Egypt	16/37 (43)
Germany	1/37 (2.7)
Italy	4/37 (11)
Norway	4/37 (11)
South Africa	1/37 (2.7)
Thailand	5/37 (14)
Uruguay	2/37 (5.4)
USA	3/37 (8.1)
Continent	n/${K}_{d}$ (%)
Africa	17/37 (46)
Asia	6/37 (16)
Europe	9/37 (24)
North America	3/37 (8.1)
South America	2/37 (5.4)
Region	n/${K}_{d}$ (%)
East Asia & Pacific	6/37 (16)
Latin America & Caribbean	2/37 (5.4)
Middle East & North Africa	16/37 (43)
North America	3/37 (8.1)
Sub–Saharan Africa	1/37 (2.7)
Western Europe	9/37 (24)
Economic classification	n/${K}_{d}$ (%)
High income	14/37 (38)
Lower middle income	16/37 (43)
Upper middle income	7/37 (19)

^a^Mean (SD); n/${K}_{d}$ (%) where *n* = HBoV-positive sample, ${K}_{d}$ = sample sources

The ranked correlation and bivariate relationship between crude prevalence of HBoV in environmental matrices and SDG 3.6.1, regional, and study related data is presented in Table S1. HBoV crude prevalence had significant positive correlation with $W{W}_{p}$ (r = 0.35, *p* = 0.034), $W{W}_{c}$ (r = 0.32, *p* = 0.057), $W{W}_{t}$ (r = 0.34, *p* = 0.043), and $W{W}_{r}$ (r = 0.34, *p* = 0.041). In addition, there is a significant direct association between HBoV positivity with sample size (r = 0.77, *p* = 1.98 × 10–8), and between population density and sample size (r = 0.37, *p* = 0.024).

### Global HBoV Prevalence in Environmental Matrices

**Fig. 2:**
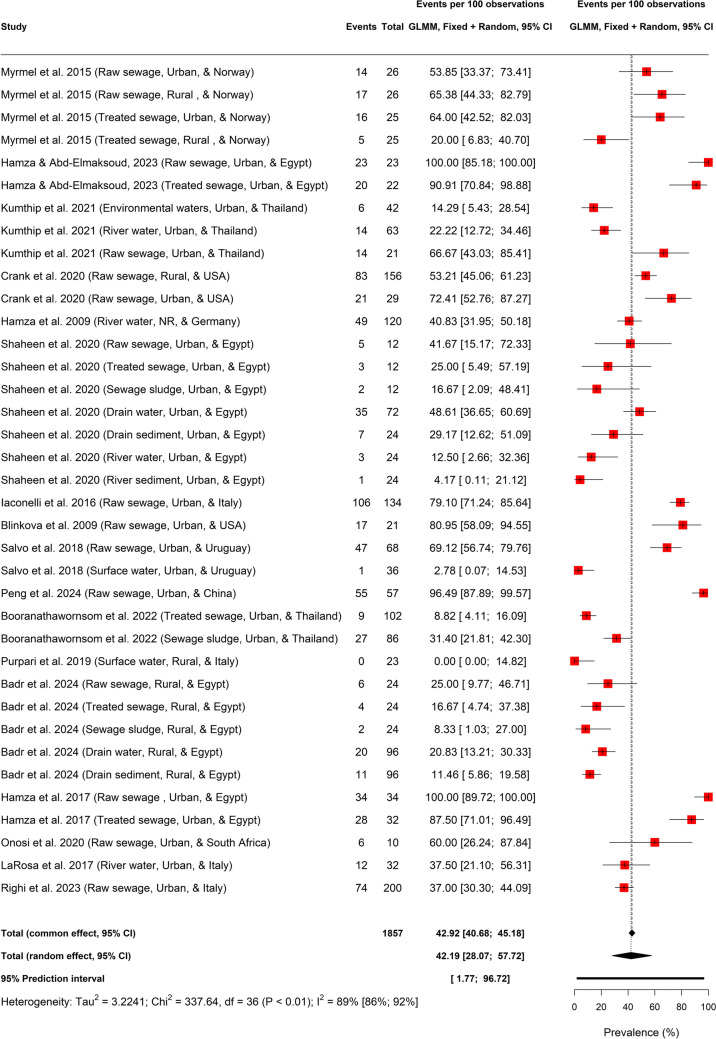
Global prevalence of HBoV in sewage and environmental matrices based on primary data of HBoV prevalence from 1857 samples

The global prevalence of HBoV in environmental waters and matrices is presented as 42.19% (95%CI: 28.07–57.72; *k* = 37; ${I}^{2}$ = 89.3% (86.3–91.7) with a 95% prediction interval (PI) of 1.77 to 96.72% (Fig. 2). Upon cross–validation, the HBoV global prevalence in environmental matrices had a robust value of 38.05% (29.79–47.07%; ${I}^{2}$ = 81%, *p* < 0.01) and a PI value of 10.55 to 76.19% (Fig. S2).

### HBoV Regional and Locational Prevalence in Environmental Milieu

**Fig. 3:**
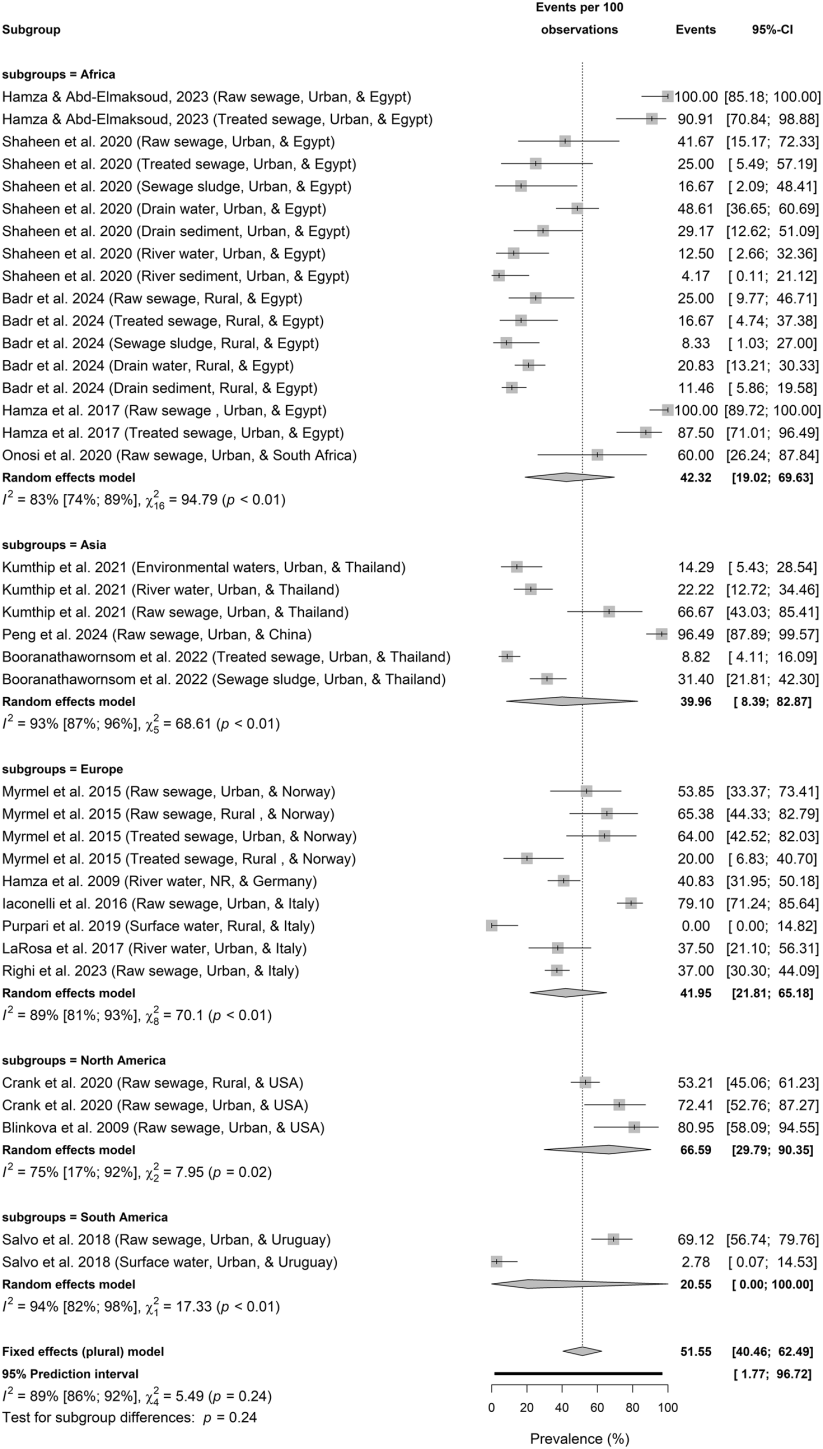

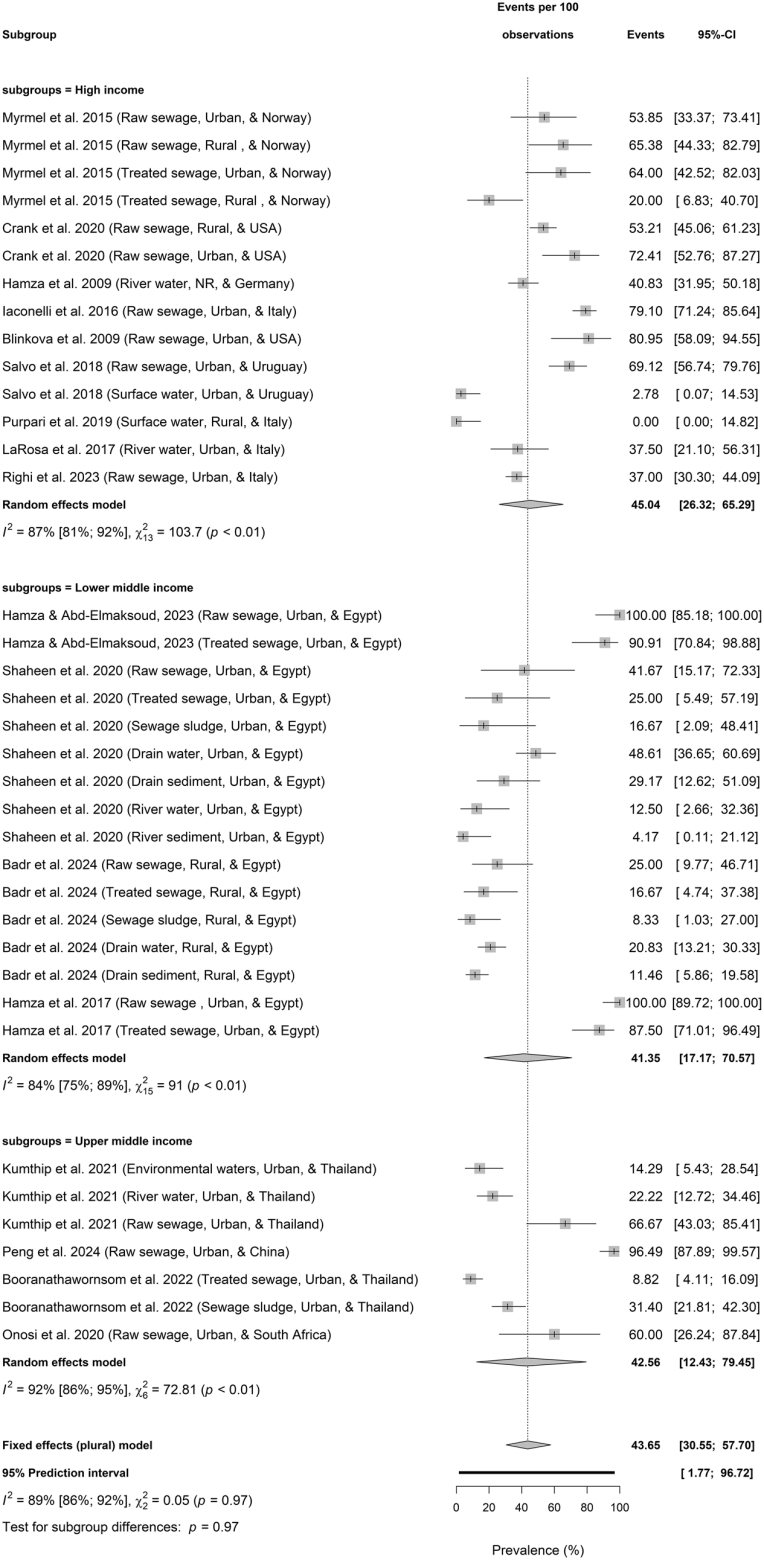

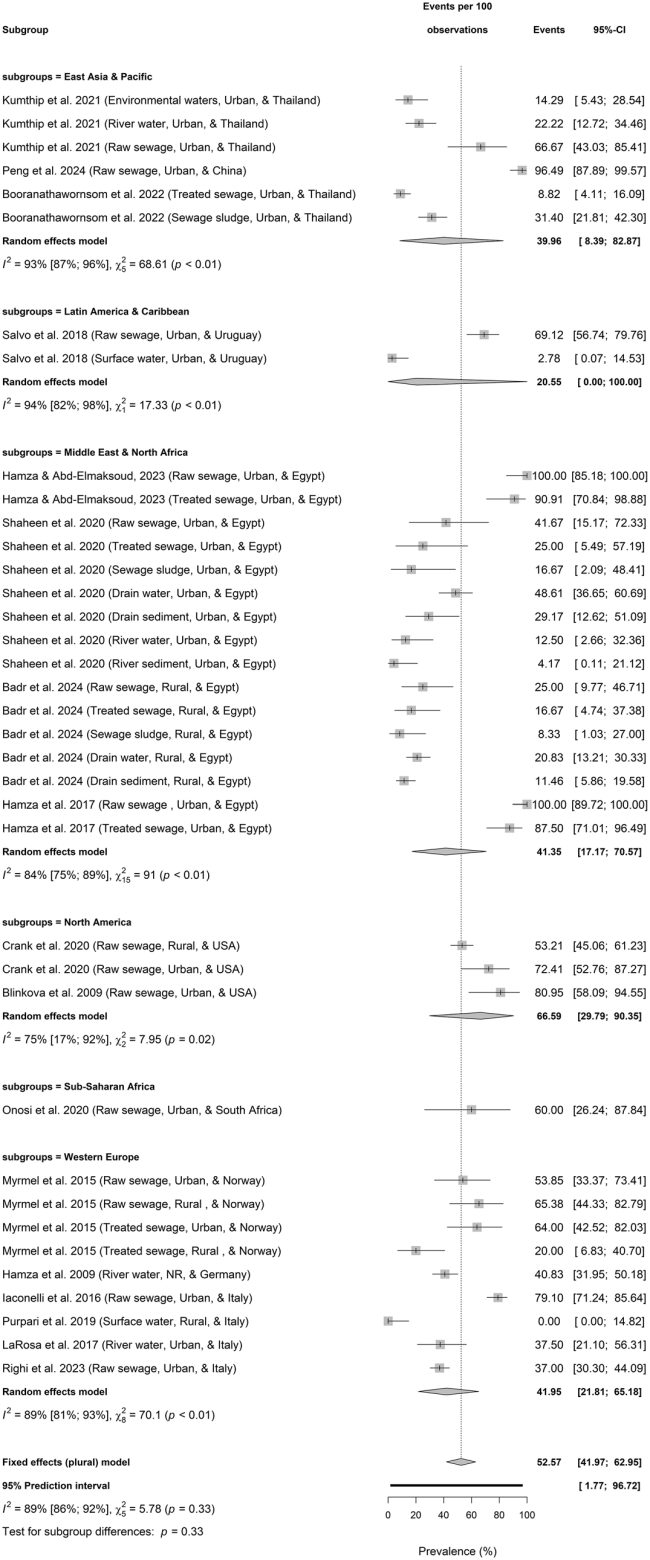

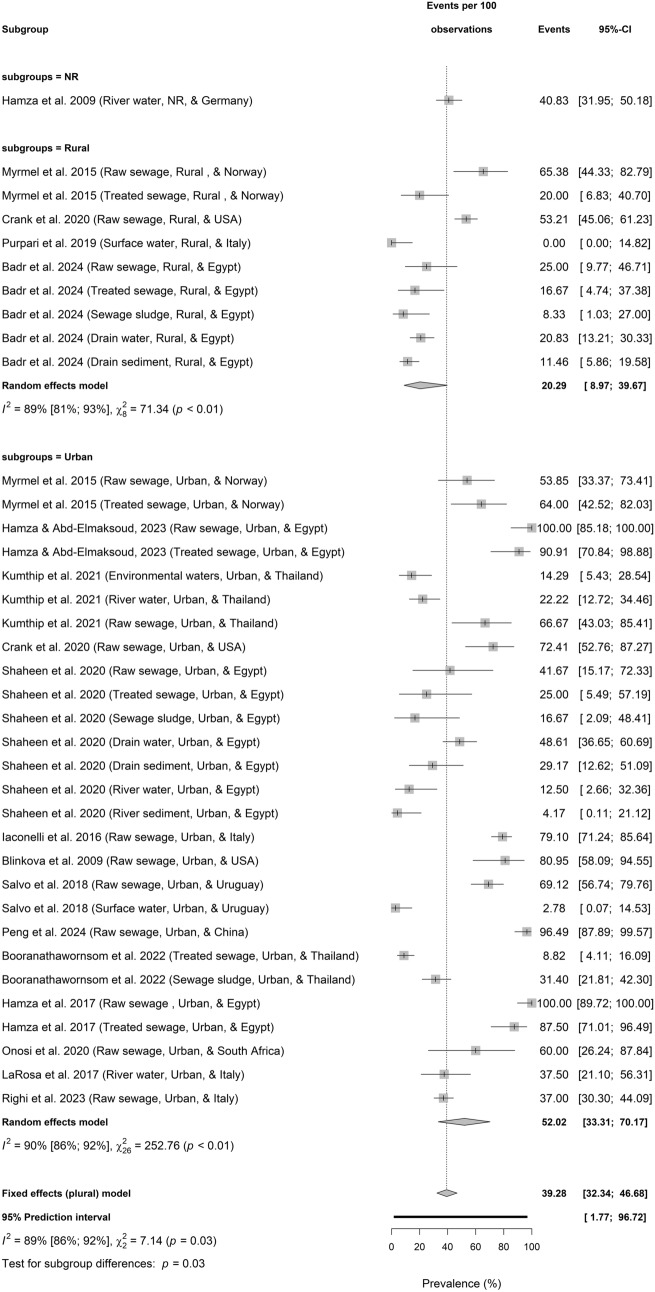
**a** Prevalence of HBoV in sewage and environmental matrices across continent based on primary data of HBoV prevalence from 1857 samples. **b** Prevalence of HBoV in sewage and environmental matrices according to economic classification based on primary data of HBoV prevalence from 1857 samples. **c** Prevalence of HBoV in sewage and environmental matrices across WHO regions based on primary data of HBoV prevalence from 1857 samples. **d** Prevalence of HBoV in sewage and environmental matrices according to locational settings based on primary data of HBoV prevalence from 1857 samples

Figures 3a–d present the regional and setting-based prevalence of HBoV in environmental water environments.

### HBoV Prevalence in Environmental Matrices Across Different Continents

Based on continents (Test for continent differences: *p* = 0.24), HBoV in water environments was highest in North America (66.59%, 29.79–90.35; ${I}^{2}$ = 75%, 17–92, *p* = 0.02; *k* = 3), followed by Africa (42.32%, 19.02–69.63; ${I}^{2}$ = 83%, 74–89, *p* < 0.01; *k* = 17), Europe (41.95%, 21.81–65.18, ${I}^{2}$ = 89%, 81–93, *p* < 0.01; *k* = 9), Asia (39.96%, 8.39–82.87; ${I}^{2}$ = 93%, 87–96, *p* < 0.01; *k* = 6), and least in South America (20.55%, 0.00–100.00; ${I}^{2}$ = 94%, 82–98, *p* < 0.01; *k* = 2) (Fig. 3a).

### HBoV Prevalence in Environmental Matrices Across Different Economic Classifications

HBoV prevalence in environmental matrices did not varied significantly according to economic classification (Test for economic classification differences: *p* = 0.97) with a value of 45.04% (26.32–65.29; ${I}^{2}$ = 87%, 81–92, *p* < 0.01; *k* = 14) in high-income countries, followed by 42.56% (12.43–79.45; ${I}^{2}$ = 92%, 86–95, *p* < 0.01, *k* = 7) in upper-middle-income nations, and 41.35% (17.17–70.57; ${I}^{2}$ = 84%, 75–89, *p* < 0.01; *k* = 16) in the lower-middle-income nations.

### HBoV Prevalence in Environmental Matrices Across Different WHO Regions

Among WHO regions, the Western Europe had highest pooled HBoV prevalence in environmental waters (41.95%, 21.81–65.18, ${I}^{2}$ = 89%, 81–93, *p* < 0.01; *k* = 9), followed by Middle East & North Africa (41.35%, 17.17–70.57; ${I}^{2}$ = 84%, 75–89, *p* < 0.01; *k* = 16), East Asia & Pacific (39.96%, 8.39–82.87; ${I}^{2}$ = 93%, 87–96, *p* < 0.01; *k* = 6), North America (66.59%, 29.79–90.35; ${I}^{2}$ = 75%, 17–92, *p* = 0.02; *k* = 3), and Latin America & Caribbean 20.55% (0.00–100.00; ${I}^{2}$ = 94%, 82–98, *p* < 0.01; *k* = 2). The single individual study from the Sub − Saharan Africa reported HBoV prevalence of 60.00% (26.24–87.84; *k* = 1) in environmental waters. Generally, HBoV prevalence in environmental waters is not significantly different among WHO regions (Test for WHO regional differences: *p* = 0.33).

### HBoV Prevalence in Environmental Matrices in Urban Versus Rural Settings

However, HBoV prevalence in environmental matrices was significantly different (test for setting differences: *p* = 0.03) between settings with 52.02% (33.31–70.17; ${I}^{2}$ = 90%, 86–92, *k* = 27, *p* < 0.01) in the urban area versus 20.29% (8.97–39.67; ${I}^{2}$ = 89%, 81–93, *p* < 0.01; *k* = 9) in the rural locations.

### HBoV Prevalence in Environmental Milieu in Different Sample Types

**Fig. 4:**
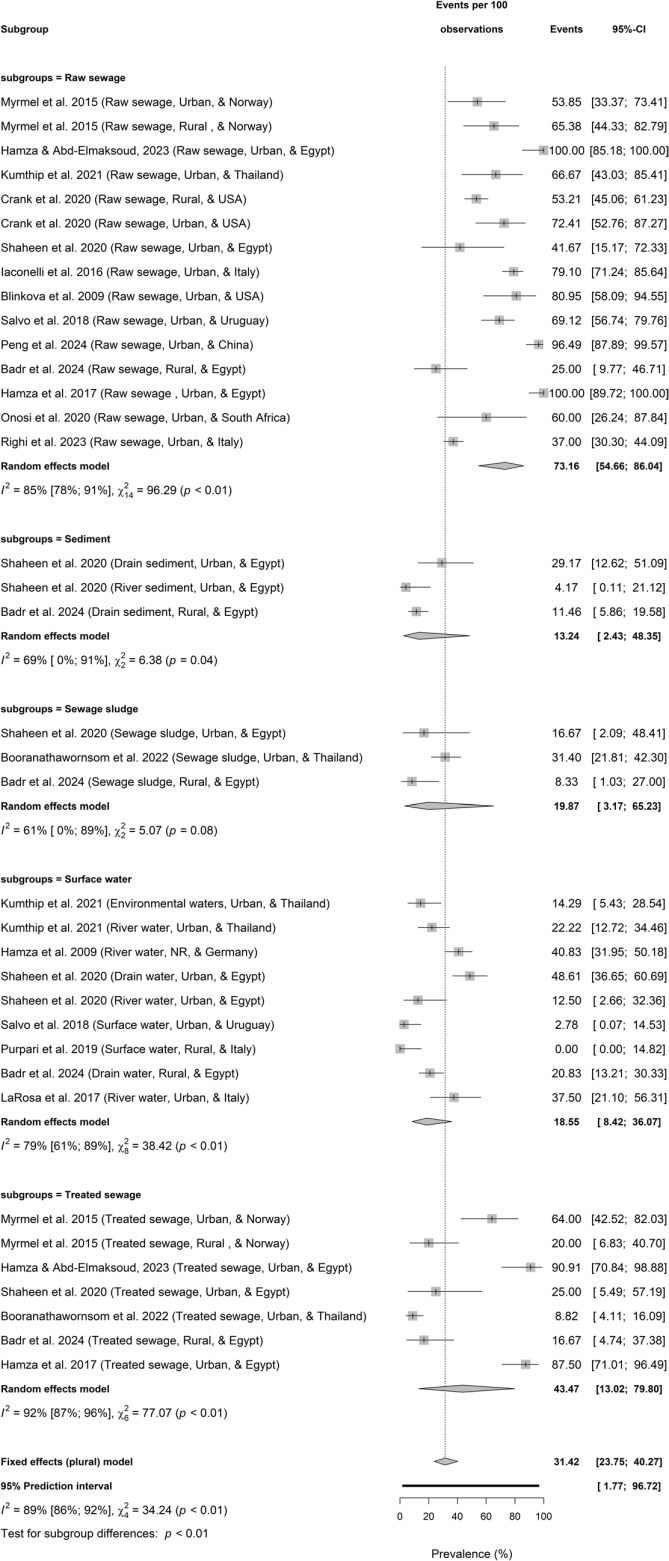
Prevalence of HBoV in sewage and environmental matrices by types based on primary data of HBoV prevalence from 1857 samples

Figure 4 shows pooled HBoV prevalence in different environmental water matrices. HBoV prevalence was significantly differences among the 5 samples (test for sample type differences: *p* < 0.01; ${I}^{2}$ = 89%, 86–92). It was 73.16% (54.66–86.04; ${I}^{2}$ = 85%, 78–91, *k* = 15, *p* < 0.01) in raw sewage versus 43.47% (13.02–79.80; ${I}^{2}$ = 92%, 87–96, *k* = 7, *p* < 0.01) in treated sewage; 19.87% (3.17–65.23, ${I}^{2}$ = 61%, 0–89, *k* = 3, *p* = 0.08)in sewage sludge versus 13.24% (2.43–48.35; ${I}^{2}$ = 69%, 0–91, *k* = 3, *p* = 0.04) in sediment; and 18.55% (8.42–36.07; ${I}^{2}$ = 79%, 61–89, *k* = 9, *p* < 0.01) in surface waters.

### Sample Methods, Concentrations, DNA Extraction, Process Controls, and Detection

Composite sampling returned HBoV pooled prevalence in environmental waters and sewage as 55.17% (32.11–76.20; ${I}^{2}$ = 75%, 39–90, *p* < 0.01; *k* = 5) and as 39.79% (23.08–59.28; ${I}^{2}$ = 90%, 87–93, *p* < 0.01; *k* = 30) by grab sampling (Fig. S3). However, the test for sampling method differences was not significant (*p* = 0.14). Also, the test for sample concentration methods differences was significantly different (*p* < 0.01) among 14 methods with substantial level of heterogeneity (${I}^{2}$ = 89%, 86–92, *p* < 0.01) (Fig. S4) signifying different capability or efficiencies in the recovery of HBoV in environmental waters and sewage. Similar, HBoV prevalence in environmental waters and sewage showed significant different among 9 types of kits/systems used for DNA extraction (*p* < 0.01) with important level of heterogeneity (${I}^{2}$ = 89%, 86–92, *p* < 0.01) (Fig. S5).

Process control in the recovery/extraction of HBoV DNA from environmental waters and sewage had substantial influence (*p* < 0.01) on assessing HBoV prevalence in the matrices although with elevated level of heterogeneity (${I}^{2}$ = 89%, 86–92, 7 process control groups, *p* < 0.01; Fig. S6). the use of murine norovirus, Mengovirus, known viral positive sample, Simian rotavirus − human adenovirus type 2, and human adenovirus type 2 as process control resulted in HBoV prevalence of 85.71% (56.57– 96.50; ${I}^{2}$ = 83%, 69–91, *k* = 8, *p* < 0.01), 32.76% (5.16–81.34; ${I}^{2}$ = 66%, 12–87, *k* = 5, *p* = 0.02), 26.98% (11.96–50.11, ${I}^{2}$ = 88%, 77–94, *k* = 6, *p* < 0.01), 23.30% (10.92–42.97; ${I}^{2}$ = 69%, 31–86;*k* = 7, *p* < 0.01), and 16.17% (10.17–24.75; ${I}^{2}$ = 25%, 0–70, *k* = 5, *p* = 0.26), respectively. Pepper mild mottle virus is seldom used as process control for HBoV recovery in environmental water matrices but in a particular case achieved HBoV prevalence of 96.49% (87.89–99.57, *k* = 1).

The HBoV pooled prevalence in sewage and environmental waters was not significantly affected by the two common detection methods employed (Test for detection method differences: *p* = 0.14; ${I}^{2}$ = 89%, 86–92; Fig. S7). While nested PCR resulted to HBoV pooled prevalence of 33.29% (23.58–44.6; ${I}^{2}$ = 90%, 86–92, *k* = 27, *p* < 0.01), real − time PCR recorded a higher pooled HBoV prevalence estimate (73.83%, 17.42–97.42; ${I}^{2}$ = 89%, 82–93, *k* = 10, *p* < 0.01).

### HBoV Circulating Genotypes/Subtypes in Sewage and Environmental Matrices

**Fig. 5:**
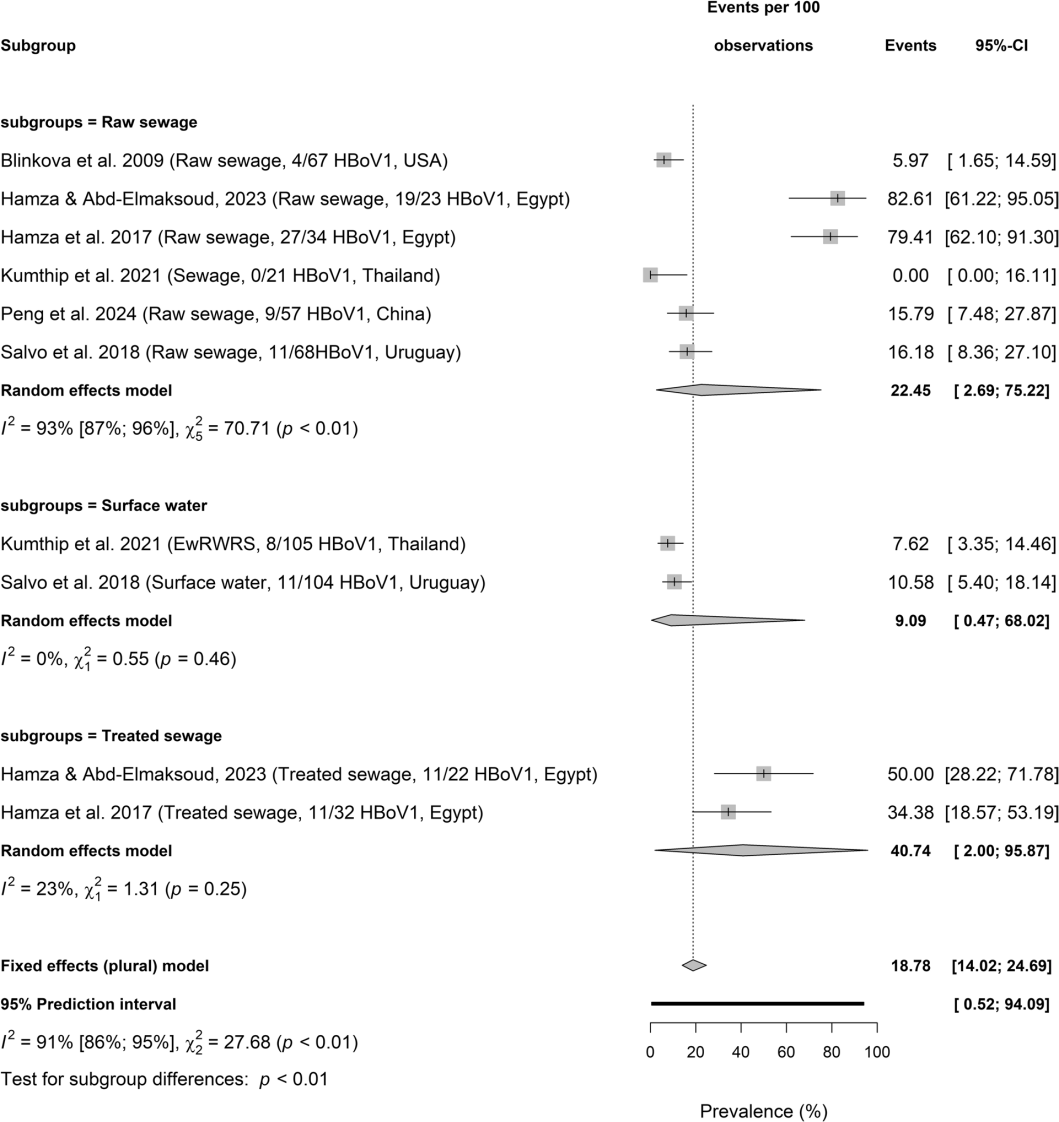

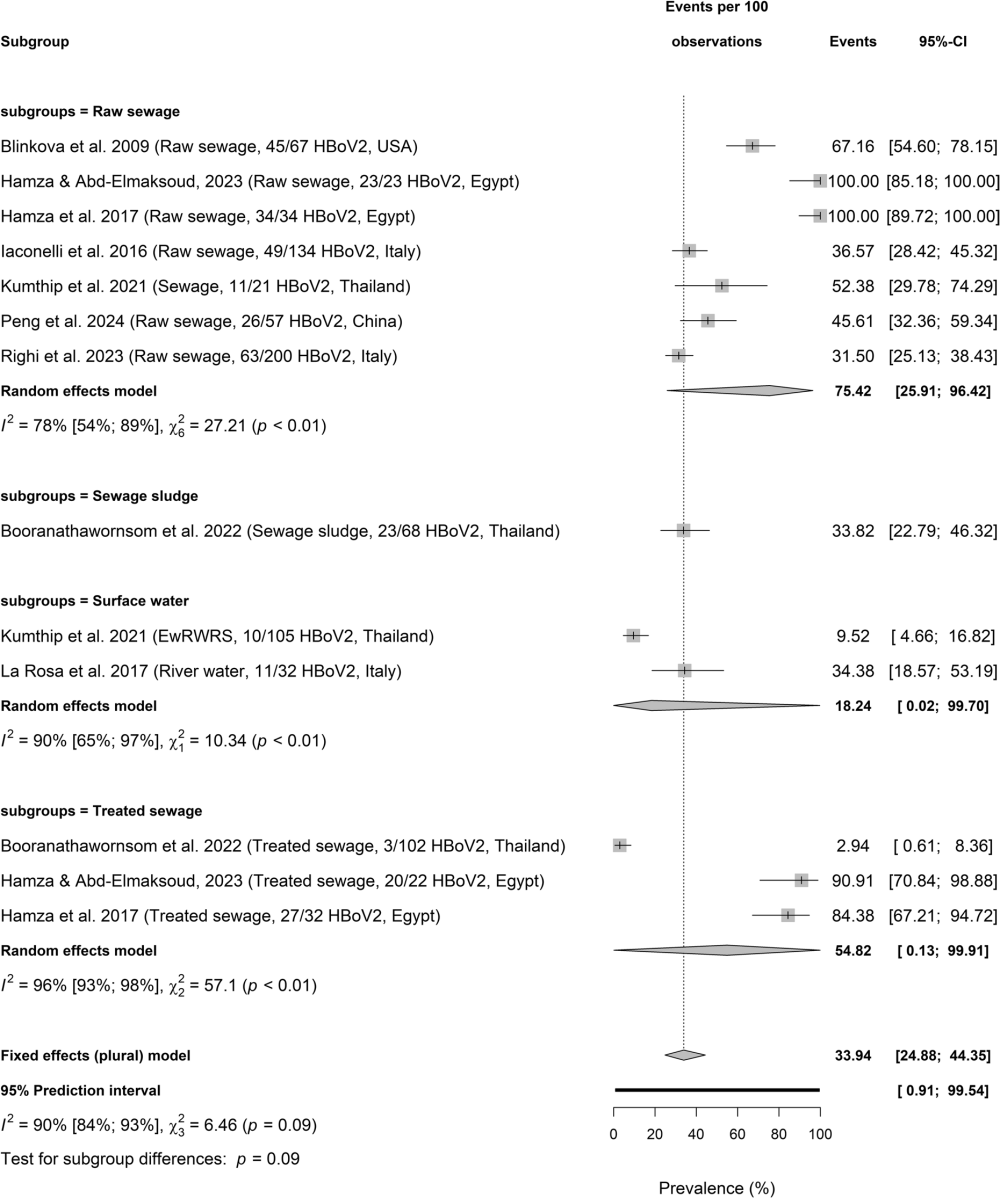

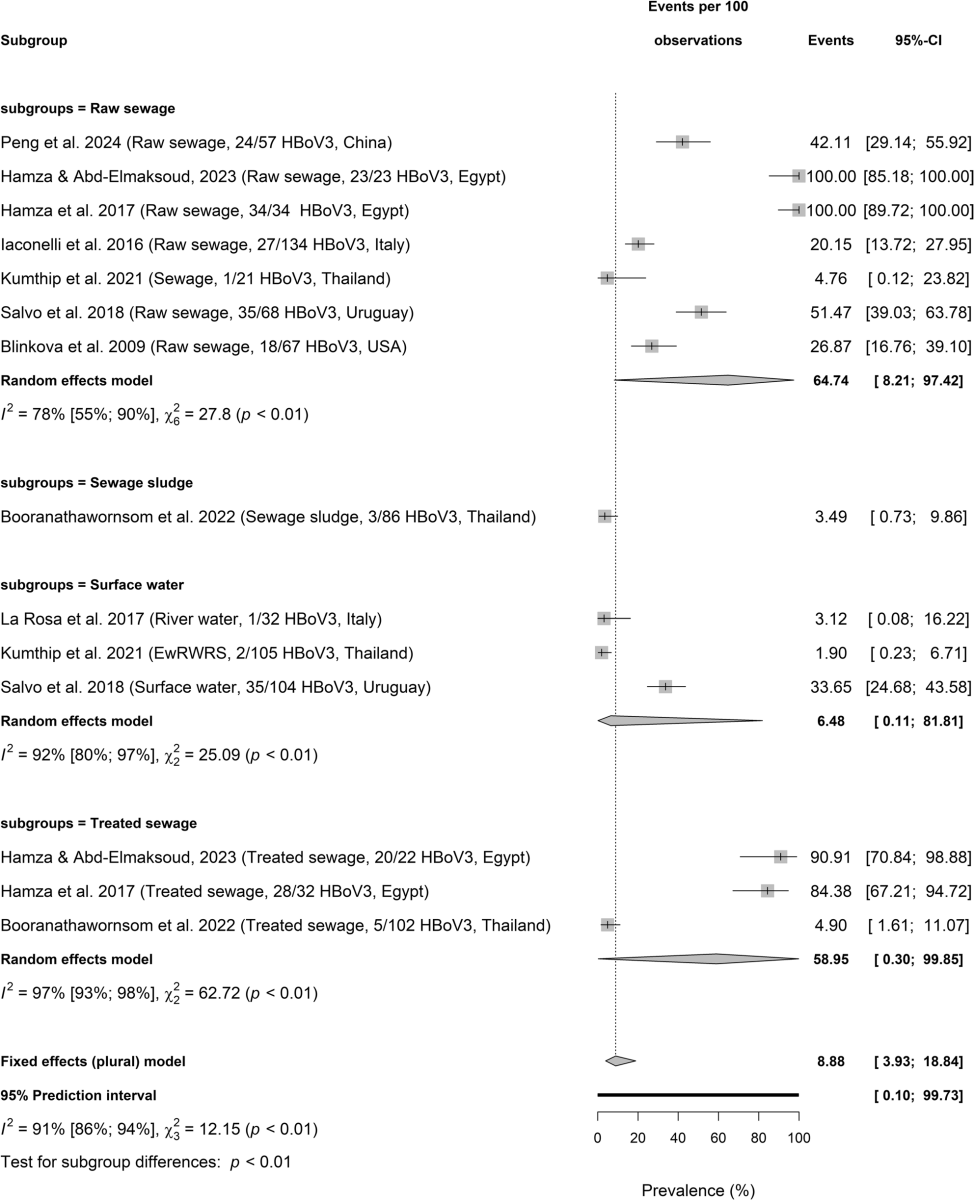
Prevalence of HBoV subtype circulating sewage and environmental matrices. **a** Prevalence of HBoV1 circulating sewage and environmental matrices based on primary data of HBoV prevalence from 1857 samples. **b** Prevalence of HBoV2 circulating sewage and environmental matrices based on primary data of HBoV prevalence from 1857 samples. **c** Prevalence of HBoV3 circulating sewage and environmental matrices based on primary data of HBoV prevalence from 1857 samples

Figure 5a, b, and c presents HBoV subtypes including HBoV1, HBoV2, and HBoV3 circulating in wastewater. HBoV1 prevalence in different samples is significantly different among 3 sample types (Test for subgroup differences: *p* < 0.01; ${I}^{2}$ = 91%, 86–95, 3 sample types, *p* < 0.01). HBoV1 pooled prevalence was highest in raw sewage (22.45%, 2.69–75.22], ${I}^{2}$ = 93%, 87–96, *k* = 6, *p* < 0.01), followed by treated sewage (40.74%, 2.00–95.87; ${I}^{2}$ = 23%, *k* = 2, *p* = 0.25) and surface water (9.09%, 0.47–68.02, ${I}^{2}$ = 0%, *k* = 2, *p* = 0.46). Whereas HBoV2 pooled prevalence in sewage and environmental matrices were not significantly different among the 4 sample types (Test for subgroup differences: *p* = 0.09; ${I}^{2}$ = 90%, 84–93), raw sewage (75.42%, 25.91–96.42, ${I}^{2}$ = 78%, 54–89, *k* = 7, *p* < 0.01) had the highest value, followed by surface water (18.24%, 0.02–99.70; ${I}^{2}$ = 90%, 65–97, *k* = 2, *p* < 0.01), and treated sewage (54.82%, 0.13–99.91, ${I}^{2}$ = 96%, 93–98, *k* = 3, *p* < 0.01). HBoV2 prevalence was reported as 33.82%, 22.79–46.32 in a single study on sewage sludge.

The pooled prevalence of HBoV3 was significantly different in 4 environmental matrices (Test for subgroup differences: *p* < 0.01) with important level of heterogeneity (${I}^{2}$ = 91% [86%; 94%], 4 sample types, *p* < 0.01). While the pooled prevalence of HBoV3 was 64.74% (8.21–97.42; ${I}^{2}$ = 78%, 55–90, *k* = 7, *p* < 0.01) in raw sewage, treated sewage had 58.95% (0.30–99.85; ${I}^{2}$ = 97%, 93–98,*k* = 3, *p* < 0.01). Also, HBoV3 prevalence was 6.48% (0.11–81.81, ${I}^{2}$ = 92%, 80–97, *k* = 3, *p* < 0.01) in surface water and 3.49% (0.73–9.86) in sewage sludge.

**Fig. 6:**
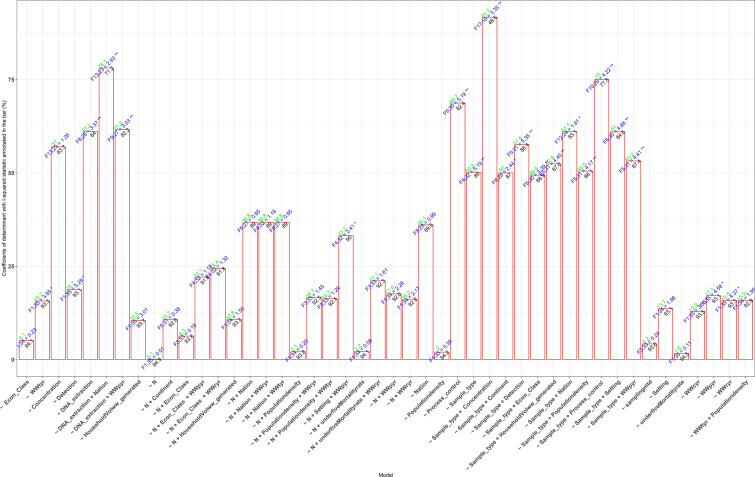
Moderating factors influencing HBoV prevalence in sewage and environment matrices based on primary data of HBoV prevalence from 1857 samples

### Factors and Interactions Associated with HBoV Prevalence in Sewage and Other Environmental Matrices

The moderating influences of distinct factors on the HBoV prevalence in environmental water is presented in Fig. 6 and Table S2. The prevalence of HBoV in sewage and environmental matrices had significant direct univariate association with Process control (${F}_{6;30}$ = 5.1937; *p* = 0.001), DNA extraction (${F}_{8;28}$ = 3.3733; *p* = 0.001), sample type (${F}_{4;32}$ = 5.1926; *p* = 0.002), Detection (${F}_{1;35}$ = 5.2797; *p* = 0.0110), $W{W}_{p}$ (${F}_{1;2}$= 35 = 4.5822; *p* = 0.025), and $W{W}_{r}$ (${F}_{1;2}$= 35 = 4.3735; *p* = 0.033), $W{W}_{t}$ (${F}_{1;35}$ = 3.9517; *p* = 0.0390), and $W{W}_{c}$ (${F}_{1;35}$ = 3.3510; *p* = 0.054) accounting for 68.67, 61.08, 50.18, 18.73,17.13, 15.79,15.65, and 12.92% of the true variance in the HBoV pooled prevalence estimate. Also, HBoV prevalence had significant relation with bivariate/multivariate additive interaction between DNA extraction and Nation (${R}^{2}$ = 78.07%, ${F}_{13;23}$ = 2.9325; *p* = 0.004), DNA extraction and $W{W}_{p}$ (${R}^{2}$ = 61.57%, ${F}_{9;27}$ = 3.0337; *p* = 0.003), sample size (N), setting and $W{W}_{p}$ (${R}^{2}$ = 33.15%, ${F}_{4;32}$ = 2.4094; *p* = 0.037), sample type and concentration method (${R}^{2}$ = 91.74%, F17;19 = 3.3542; *p* = 0.002), sample type and continent (${R}^{2}$ = 49.97%, ${F}_{8;28}$ = 2.4380; *p* = 0.018), sample type and detection method (${R}^{2}$ = 57.59%, ${F}_{5;31}$ = 5.3541; *p* = 0.001), sample type and economic classification (${R}^{2}$ = 49.41%, ${F}_{6;30}$ = 3.3901; *p* = 0.006), sample type and Household vol. sewage generated (${R}^{2}$ = 52.35%, ${F}_{5;31}$ = 4.3997; *p* = 0.001), sample type and nation (${R}^{2}$ = 61.14%, ${F}_{12;24}$ = 1.9073; *p* = 0.041), sample type and population density (50.47%, ${F}_{5;31}$ = 4.1740; *p* = 0.002), sample type and process control (${R}^{2}$ = 75.0%, ${F}_{10;26}$ = 4.2216; *p* = 0.002), sample type and setting (${R}^{2}$ = 61.03%, ${F}_{6;30}$ = 4.6748; *p* = 0.002), and sample type and $W{W}_{p}$ (${R}^{2}$ = 53.21%, ${F}_{5;31}$ = 4.4139; *p* = 0.002) accounting for varying degrees of true variance in the prevalence estimates.

## Discussion

Environmental matrices contribute greatly to the persistence and transmission fluxes or patterns of waterborne pathogens including HBoV. The current investigation prototypically estimated the global and regional prevalence of HBoV and its genotypes in environmental waters. Research interest or data on the presence of HBoV sparsely distributed global-wise with few data from Egypt, Italy, Thailand, USA, China, Ecuador, Germany, Norway, South Africa, Spain, and Uruguay (Fig. 1). Although clinical manifestations of HBoV in respiratory and diarrhoeic events are more commonly reported across developed and developing nations (Allander et al., 2005; Fry et al., 2007; Lau et al., 2007; Pozo et al., 2007; Vicente et al., 2007; Campe et al., 2008; Arthur et al., 2009; Chow et al., 2010; Chhabra et al., 2013; Broccolo et al., 2015; Guido et al., 2016; Lee et al., 2016; Ong et al., 2016; Qiu et al., 2017; Rikhotso et al., 2018; Bergallo et al., 2019), it is not clear why the surveillance of HBoV in environmental milieu has not receive deserved attention.

Findings from the current study revealed the grand average sample size of the environmental waters as $50.19\pm 44.99$ per 37 matrix type. Larger sample sizes are generally known to improve the precision of effect size estimates while smaller sample sizes are inundated with lack of precision and higher statistical uncertainties (Slavin & Smith, 2009). Also, HBoV was detected in raw sewage, surface water, treated sewage, sediment, and sewage sludge through grab, composite, or swab sampling techniques. The need to assay environmental waters such as lakes, dams, ponds, estuaries, salt waters, and marine waters cannot be overemphasized. For instance, the reported contamination of shellfish which are estuarine and marine animals with HBoV (La Rosa et al., 2018; Purpari et al., 2019; Onosi et al., 2020; Kumthip et al., 2021; Nascimento et al., 2022) is an indication of not only HBoV presence in the aquatic milieu, but it may also suggest point source/diffusion source pollution of the environments from sewage and other anthropogenic pollutions with subsequent ecological impacts.

Diverse HBoV concentration methods were reported in the studies (Tables 1 and 2). The diversity could be attributed to the lack of an official standard ISO/WHO method for specifically concentrating HBoV in food and environmental sources. Previous studies have highlighted the significance of concentration methods on the recovery or quantification of viruses such as adenovirus and norovirus in environmental waters including wastewater (Maunula et al., 2009) and it dependent on the viral targets (Crank et al., 2020). Thus, future investigations are needed to assess comparative efficiencies of the various concentration techniques in recovering or quantifying HBoV in different environmental matrices to establish the best-buys or best fit methods in different scenarios. The performance of glycine-CF and PEG-dextran concentration methods on HBoV in wastewater have been reported (Crank et al., 2020). The glycine-CF method has the merit of short-turnaround time and requires a low volume of sample (which is meritorious for limited samples), but PEG-dextran method, though a time-consuming and backbreaking method compared to the glycine-CF method is the WHO’s endorsed standard concentration method for recovering most viruses from sewage (Crank et al., 2020).

Like the concentration methods, HBoV particles concentration process controls were achieved by utilizing different virus control systems. It is lacking whether these viruses have comparable characteristics in environmental matrices with HBoV or not. Incomparable or differing behavior of the viral controls and HBoV in environmental milieu would result in incorrect estimate or recovery inefficiency. While the present study highlighted that HBoV was detected in the environmental samples via PCR-based techniques, 73% of the environmental matrices were sampled from urban settings compared with 24% from rural settings. Viral cultivation, plaque assay, cell culture and serological/antigen–antibody methods, mostly used to assess infectiousness of viral particles were not reported. This is due to lacks or limited success of viral culture, cultivation and serological/antigen–antibody techniques for HBoV (Rikhotso et al., 2018). Also, the observed data depicted an elevated level of inequality between urban and rural areas in term of HBoV monitoring programs. The disparity among dwelling settings are not surprising. Efforts should be made to ensure equality in the implementation of environmental surveillance with the intentional inclusion of rural and pre-urban areas.

The present study observed a significant direct and moderate positive relationship between crude prevalence of HBoV in environmental matrices and SDG 3.6.1 variables (Fig. S1b). This suggests that wastewater works contribute to some extent to the persistence and dissemination dynamics of HBoV in the environment. It may also disclose occurrence and continuous shedding of HBoV among the general population either by symptomatic or asymptomatic individuals. In addition, there is a significant direct association between HBoV positivity with sample size, and between population density and sample size. Thus, for accurate estimate of HBoV in environmental matrices, large samples are required as previously mentioned that smaller samples lead to imprecision and higher uncertainties in effect estimate (Slavin & Smith, 2009).

The global prevalence of HBoV in environmental matrices was 42.19% which upon cross-validation remain stable/robust as 38.05%. This represents a substantial presence and persistence of HBoV in the environment and call for an integrated approach in countering its transmission to humans. Some individual studies found a lower value of HBoV prevalence in environmental matrices than the global estimate ranging from 0 to 2.8% in surface water (Salvo et al., 2018; Purpari et al., 2019), 8.8–20.0% in treated sewage effluents (Myrmel et al., 2015; Booranathawornsom et al., 2022; Badr et al., 2024), 11.5–29.2% in drain water/sediment (Shaheen et al., 2020; Badr et al., 2024), 25–41.7% in raw sewage (Shaheen et al., 2020; Righi et al., 2023; Badr et al., 2024), 4.2–40.8% in river water and river sediment (Hamza et al., 2009; La Rosa et al., 2017; Shaheen et al., 2020; Kumthip et al., 2021), 8.3–31.4% in sewage sludge (Shaheen et al., 2020; Booranathawornsom et al., 2022; Badr et al., 2024) whereas others found higher values including 48.6% in drain water (Shaheen et al., 2020), 95.6% in mixed sewage (Hamza & Abd-Elmaksoud, 2023), 53.2–100% in raw sewage (Blinkova et al., 2009; Myrmel et al., 2015; Iaconelli et al., 2016; Hamza et al., 2017; Salvo et al., 2018; Crank et al., 2020; Onosi et al., 2020; Kumthip et al., 2021; Peng et al., 2024), and 64–87.5% in treated sewage (Myrmel et al., 2015; Hamza et al., 2017).

The prevalence of HBoV lack any discernible differences across continents, economic classification, and WHO regional grouping (Figs. 3a–d). This implies the ubiquity and worldwide presence and persistence of HBoV in the environment.

However, there was an observable significant difference in HBoV prevalence in the urban (52.02%) versus rural (20.29%) environments probably revealing disparity in HBoV monitoring and anthropogenic pollutions between the two settings. Also, HBoV prevalence was significantly differences among the sample types (Fig. 4). Several factors contribute to the differences including variations in population size, sanitation/hygiene, wastewater generated, treated, and discharged into the environments to mention a few. HBoV prevalence reported in rural setting in raw sewage ranged from 25 to 65.4% in Norway, USA, and Egypt (Myrmel et al., 2015; Crank et al., 2020; Badr et al., 2024), 11.5% in sediment in Egypt (Badr et al., 2024), 8.3% in sewage sludge in Egypt (Badr et al., 2024), 0–40.8% in surface water from Egypt, Germany, and Italy (Hamza et al., 2009; Purpari et al., 2019; Badr et al., 2024), and 16.7–20.0% in treated sewage from Egypt and Norway (Myrmel et al., 2015; Badr et al., 2024). Previous studies reported HBoV prevalence in urban raw sewage as 53.8% in Norway (Myrmel et al., 2015), 41.7–100% in Egypt (Hamza et al., 2017; Shaheen et al., 2020; Hamza & Abd-Elmaksoud, 2023), 66.7% in Thailand (Kumthip et al., 2021), 72.4–81% in USA (Blinkova et al., 2009; Crank et al., 2020), 69.1% in Uruguay (Salvo et al., 2018), 96.5% in China (Peng et al., 2024), 60.0% in South Africa (Onosi et al., 2020), and 37–79.1% in Italy (Iaconelli et al., 2016; Righi et al., 2023); urban sediment as 4.2–29.2% in Egypt (Shaheen et al., 2020); urban sewage sludge as 16.7% in Egypt (Shaheen et al., 2020) and 31.4% in Thailand (Booranathawornsom et al., 2022); urban surface water as 14.3–22.2% in Thailand (Kumthip et al., 2021), 12.5–48.6% in Egypt (Shaheen et al., 2020), 2.8% in Uruguay (Salvo et al., 2018), and 37.5% in Italy (La Rosa et al., 2017); urban treated sewage as 8.8% in Thailand (Booranathawornsom et al., 2022), 64.0% in Norway (Myrmel et al., 2015), and 25.0 –90.9% in Egypt (Hamza et al., 2017; Shaheen et al., 2020; Hamza & Abd-Elmaksoud, 2023).

Sampling techniques do not differ substantially in efficiency in detecting HBoV, but composite sampling return a higher prevalence rate compared to grab sampling. This suggests that grab sampling may result in false positive results due to temporal variation in HBoV concentration in environmental samples in contrast with composite sampling. Also, sample concentration methods, DNA extraction kits, and process control showed different capabilities or efficiencies in the recovery of HBoV from environmental waters and sewage with important levels of inconsistencies or non-combinability. Thus, a standardized HBoV concentration method is required as aforementioned. While HBoV detection in sewage and environmental waters was not significantly different by nested PCR and real-time PCR in the study, real − time PCR recorded higher prevalence than nested PCR. It is however believed that real-time PCR increased specificity, offers greater sensitivity, and reduce the likelihood of false positive results (Chieochansin et al., 2008).

HBoV subtypes including HBoV1, HBoV2, and HBoV3 circulating in wastewater. HBoV1 pooled prevalence was highest in raw sewage (22.45%), followed by treated sewage (40.74%) and surface water (9.09%); HBoV2 higher in raw sewage (75.42%), followed by treated sewage (54.82%), and surface water (18.24%) though not substantially different; HBoV3 with 64.74% in raw sewage, 58.95% in treated sewage, 6.48% (0.11–81.81) in surface water and 3.49% in sewage sludge. These distributions may have implication in the role of the matrices in the dissemination of HBoV subtypes. Among four known HBoV subtypes (HBoV1, 2, 3, and 4), HBoV1 is oft associated with respiratory diseases in children with acute wheezing, pneumonia, bronchiolitis, or asthma while HBoV2, 3, and 4 are oft detected in gastrointestinal infections (Kapoor et al., 2010; Jartti et al., 2012; Iaconelli et al., 2016).

Several factors affect HBoV detection directly and significantly in environmental matrices including $W{W}_{p}$, $W{W}_{r}$, $W{W}_{t}$, $W{W}_{c}$, viral nucleic acid extraction process control, DNA extraction, sample type, and detection method accounting for 12.92–68.67% true variance in the HBoV prevalence. Also, bivariate/multivariate additive interaction among the factors in some cases, explained 33.15–91.74% true variance in the prevalence estimates. Thus, they could be harnessed individually or in combination in a few ways to break the chain of transmission of HBoV in the environment or optimize its detection.

The infectivity of HBoV from environmental matrices are unknown. There is a need to develop suitable methods to assess the infectiousness of HBoV in different environmental milieu. In addition, the relationship between coliphages, fecal indicator bacterial, and viral pathogens employed in determining microbiological compliance of sewage and environmental samples need to be established whether they are suitable surrogates or proxies for predicting HBoV presence in environmental matrices.

## Supplementary Information

Below is the link to the electronic supplementary material.

Supplementary file1 (TIFF 1044 KB)

Supplementary file2 (TIFF 1567 KB)

Supplementary file3 (TIFF 2261 KB)

Supplementary file4 (TIFF 2848 KB)

Supplementary file5 (TIFF 2765 KB)

Supplementary file6 (TIFF 2608 KB)

Supplementary file7 (TIFF 2243 KB)

Supplementary file8 (DOCX 46 KB)

## Data Availability

No datasets were generated or analyzed during the current study.

## Code Availability

The code associated with this manuscript will be made available upon reasonable request to the corresponding author.
