# Supplementary material for: Prevalence of Human Bocavirus in Sewage, Surface Waters, and Other Environmental Milieux: A Meta-regression Modelling
Source: Food Environ Virol. 2025 Jun 17;17(3):34. doi: 10.1007/s12560-025-09648-0 (PMC12174196; doi:10.1007/s12560-025-09648-0)
Supplement: Supplementary file 8 — Supplementary file8 (DOCX 46 KB) [file 12560_2025_9648_MOESM8_ESM.docx]

**Supplementary methods**

**Search strategy**

The search matrix involved:

**PubMed:** "human bocavirus*"[tiab] & (water*[tiab] | river*[tiab] | stream*[tiab] | lake*[tiab] | groundwater*[tiab] | pond*[tiab] | estuar*[tiab] | dam[tiab] | wastew*[tiab] | sewage*[tiab]) & 1800/01/01:2024/12/31[dp].

**Scopus**

Your query: ((( TITLE-ABS-KEY ( ( water* OR river* OR stream* OR lake* OR groundwater* OR pond*OR estuar* OR dam OR wastew* OR sewage *) AND "human bocavirus*") ) ) AND ( LIMIT-TO ( DOCTYPE,"ar" ) ) )

2025 excluded

**Web of science:**

Search: (TS=(( "human bocavirus*") AND (water* OR river* OR stream* OR lake* OR groundwater* OR pond*OR estuar* OR dam OR wastew* OR sewage *))) AND (DT==("ARTICLE") AND DOP=1900-01-01/2024-12-31)

**EBSCOhost**

| **Query** | **Limiters/Expanders** | **Last Run Via** |
| --- | --- | --- |
| (human baculovirus) AND ((water* OR river* OR stream* OR lake* OR groundwater* OR pond*OR estuar* OR dam OR wastew* OR sewage)) | Expanders  - Applyequivalent subjects  Search modes  -Boolean/Phrase | Interface  - EBSCOhostResearch Databases  Search Screen  - AdvancedSearch  Database  - Academic SearchUltimate;Agricola;APAPsycInfo;AtlaSerials, ReligionCollection;Business SourceUltimate;CAB Abstracts withFull Text;CINAHL with FullText;Communication & MassMedia Complete;eBookCollection(EBSCOhost);ERIC;Fish,Fisheries & Aquatic BiodiversityWorldwide;GlobalHealth;GreenFILE;HealthSource - ConsumerEdition;Health Source:Nursing/AcademicEdition;Library, InformationScience & TechnologyAbstracts;MasterFILEPremier;MasterFILE ReferenceeBook Collection;MEDLINE withFull Text;NewspaperSource;Regional BusinessNews;SocINDEX with Full with FullText;Teacher Reference CenterText;SPORTDiscus |

**Supplementary results**

Records identified from:

Databases (**N=104**):

- PubMed = 25
- WOS = 34
- Scopus = 31
- EBSCOhost = 14

Records removed *before screening*:

Duplicate records removed (n= 67)

**Identification**

**Included**

Reports assessed for eligibility

(n = 20)

Reports excluded: n = 0

Studies included in systematic review (n = 20)

Studies included in meta–analysis (n=19)

Disaggregated meta–analysis (n = 37)

Records screened by title/abstract

(**n = 36 )**

Records excluded

(n = 16)

Reports sought for retrieval

(n = 20)

Reports not retrieved

(n =0)

**Screening**

**Identification of studies via databases last updated**

**Figure S1. Flow diagram for selecting studies on HBoV prevalence in environmental matrices.** McCall et al. (2021) excluded:

Figure S2. Cross-validation of the global prevalence of HBoV in environmental waters and matrices based on primary data of HBoV prevalence from 1857 samples.

Figure S3 Prevalence of HBoV in sewage and environmental matrices by sampling methods based on primary data of HBoV prevalence from 1857 samples.

Figure S4: Prevalence of HBoV in sewage and environmental matrices by sample concentration methods based on primary data of HBoV prevalence from 1857 samples.

Figure S5: Prevalence of HBoV in sewage and environmental matrices by DNA extraction method based on primary data of HBoV prevalence from 1857 samples.

Figure S6: Prevalence of HBoV in sewage and environmental matrices by process control methods based on primary data of HBoV prevalence from 1857 samples.

Figure S7: Prevalence of HBoV in sewage and environmental matrices by detection methods based on primary data of HBoV prevalence from 1857 samples.

**Table S1: Affinity between HBoV prevalence and environmental matrices, (wastewater production (WWp), collection (WWc), treatment (WWt), and reuse (WWr), and regional covariates.**

| Bivariate correlation | R-value | p-value |
| --- | --- | --- |
| N vs population density | 0.37 | 0.02 |
| N vs WWt | 0.12 | 0.49 |
| N vs WWp | 0.09 | 0.58 |
| N vs WWc | 0.08 | 0.64 |
| N vs WWr | 0.07 | 0.70 |
| N vs PR | –0.03 | 0.87 |
| P vs N | 0.77 | 1.98 × 10^–8^ |
| P vs WWt | 0.3 | 0.07 |
| P vs WWp | 0.28 | 0.09 |
| P vs WWr | 0.27 | 0.11 |
| P vs WWc | 0.25 | 0.13 |
| PR vs WWp | 0.35 | 0.03 |
| PR vs WWr | 0.34 | 0.04 |
| PR vs WWt | 0.34 | 0.04 |
| PR vs WWc | 0.32 | 0.06 |
| WWc vs WWt | 0.99 | 5.78 × 10^–33^ |
| WWc vs WWr | 0.96 | 7.24 × 10^–22^ |
| WWc vs population density | –0.22 | 0.20 |
| WWp vs WWt | 0.99 | 3.33 × 10^–29^ |
| WWp vs WWc | 0.97 | 4.42 × 10^–24^ |
| WWp vs WWr | 0.96 | 6.47 × 10^–22^ |
| WWp vs Population density | –0.18 | 0.30 |
| WWr vs Population density | –0.09 | 0.60 |
| WWt vs WWr | 0.96 | 2.75 × 10^–20^ |
| WWt vs Population density | –0.2 | 0.24 |

N = sample size; PR = HBoV crude prevalence; P = number of HBoV positive sample.

**Table S2:** **Factors and interactions associated with HBoV prevalence in sewage and other environmental matrices**.

| Model | I^2^ | R^2^ | Test of moderators |
| --- | --- | --- | --- |
| ~ Econ_Class | 94.14% | 5.05% | F_1;34_ = 0.2276; p = 0.7920 |
| ~ WWt | 93.25% | 15.65% | F_1;35_ = 3.9517; **p = 0.0390** |
| ~ Concentration | 83.72% | 57.00% | F_13;23_ = 1.2621; p = 0.2180 |
| ~ Detection | 93.06% | 18.73% | **F_1;35_ = 5.2797; p = 0.0110** |
| ~ DNA extraction | 83.97% | 61.08% | **F_8;28_ = 3.3733; p = 0.0010** |
| ~ DNA extraction + Nation | 71.21% | 78.07% | F_13;23_ = 2.9325; p = 0.0040 |
| ~ DNA extraction + WWp | 82.19% | 61.57% | F_9;27_ = 3.0337; p = 0.0030 |
| ~ HouseholdVolww_generated | 93.70% | 10.51% | F_1;35_ = 3.0102; p = 0.0740 |
| ~ N + Continent | 92.60% | 10.69% | F_5;31_ = 0.3778; p = 0.8390 |
| ~ N + Econ_Class | 93.78% | 6.25% | F_3;33_ = 0.1886; p = 0.8870 |
| ~ N + Econ_Class + WWp | 91.93% | 22.05% | F_4;32_ = 1.1844; p = 0.2510 |
| ~ N + Econ_Class + WWr | 91.76% | 24.43% | F_4;32_ = 1.3169; p = 0.2240 |
| ~ N + HouseholdVolww_generated | 93.24% | 10.77% | F_1;34_ = 1.4997; p = 0.2070 |
| ~ N + Nation | 88.97% | 36.82% | F_9;27_ = 0.8506; p = 0.4920 |
| ~ N + Nation + WWr | 88.97% | 36.82% | F_4;32_ = 1.1844; p = 0.2860 |
| ~ N + Nation + WWt | 88.97% | 36.82% | F_9;27_ = 0.8506; p = 0.5340 |
| ~ N + population density | 93.79% | 2.07% | F_1;34_ = 0.1989; p = 0.7860 |
| ~ N + population density + WWr | 92.48% | 16.65% | F_3;33_ = 1.4460; p = 0.2050 |
| ~ N + population density + WWt | 92.44% | 16.28% | F_3;33_ = 1.2895; p = 0.2110 |
| ~ N + Setting + WWp | 90.02% | 33.15% | F_4;32_ = 2.4094; p = 0.0370 |
| ~ N + under-five mortality rate | 94.14% | 2.19% | F_1;34_ = 0.0836; p = 0.9100 |
| ~ N + under-five mortality rate + WWr | 92.30% | 21.06% | F_3;33_ = 1.6078; p = 0.1510 |
| ~ N + WWp | 92.61% | 17.58% | F_1;34_ = 2.2811; p = 0.0770 |
| ~ N + WWr | 92.79% | 16.17% | F_1;34_ = 2.1647; p = 0.0980 |
| ~ Nation | 89.55% | 36.13% | F_8;28_ = 0.9856; p = 0.4030 |
| ~ Population density | 94.22% | 1.96% | F_1;35_ = 0.3871; p = 0.4980 |
| ~ Process control | 82.37% | 68.67% | F_6;30_ = 5.1937; p = 0.0010 |
| ~ Sample type | 89.03% | 50.18% | **F_4;32_ = 5.1926; p = 0.0020** |
| ~ Sample type + Concentration | 48.60% | 91.74% | F_17;19_ = 3.3542; p = 0.0020 |
| ~ Sample type + Continent | 87.03% | 49.97% | F_8;28_ = 2.4380; p = 0.0180 |
| ~ Sample type + Detection | 86.39% | 57.59% | F_5;31_ = 5.3541; p = 0.0010 |
| ~ Sample type + Econ_Class | 88.93% | 49.41% | F_6;30_ = 3.3901; p = 0.0060 |
| ~ Sample type + HouseholdVolww_generated | 87.77% | 52.35% | F_5;31_ = 4.3997; p = 0.0010 |
| ~ Sample type + Nation | 83.07% | 61.14% | F_12;24_ = 1.9073; p = 0.0410 |
| ~ Sample type + Population density | 88.10% | 50.47% | F_5;31_ = 4.1740; p = 0.0020 |
| ~ Sample type + Process control | 77.83% | 75.00% | F_10;26_ = 4.2216; p = 0.0020 |
| ~ Sample type + Setting | 84.60% | 61.03% | F_6;30_ = 4.6748; p = 0.0020 |
| ~ Sample type + WWp | 87.36% | 53.21% | F_5;31_ = 4.4139; p = 0.0020 |
| ~ sampling method | 93.76% | 4.20% | F_3;33_ = 0.2407; p = 0.8770 |
| ~ Setting | 93.07% | 13.68% | F_1;34_ = 1.9624; p = 0.1310 |
| ~ under five Mortalit yrate | 94.45% | 1.56% | F_1;35_ = 0.1111; p = 0.7370 |
| ~ WWc | 93.45% | 12.92% | F_1;35_ = 3.**3510; p = 0.0540** |
| ~ WWp | 93.14% | 17.13% | F_1;2_= 35 = 4.5822; **p = 0.0250** |
| ~ WWr | 93.29% | 15.79% | F_1;2_= 35 = 4.3735**; p = 0.0330** |
| ~ WWt + Population density | 92.93% | 15.92% | F_1;34_ = 1.9496; p = 0.1170 |
| ~ N | 94.31% | 0.08% | F_1;2_= 35 = 0.0120; p = 0.9150 |

Econ_Class: economic classification; HouseholdVolww_generated: Household volume of wastewater generated; Wastewater production (WWp), collection (WWc), treatment (WWt), and reuse (WWr).
